# Enrichment of oligodendrocyte precursor phenotypes in subsets of low-grade glioneuronal tumours

**DOI:** 10.1093/braincomms/fcae156

**Published:** 2024-05-06

**Authors:** Zejun Duan, Jing Feng, Yuguang Guan, Shouwei Li, Bin Wu, Yang Shao, Zhong Ma, Zejuan Hu, Lei Xiang, Mingwang Zhu, Xiaolong Fan, Xueling Qi

**Affiliations:** Department of Pathology, Sanbo Brain Hospital, Capital Medical University, Beijing 100093, China; Department of Pathology, Sanbo Brain Hospital, Capital Medical University, Beijing 100093, China; Department of Neurosurgery, Sanbo Brain Hospital, Capital Medical University, Beijing 100093, China; Department of Neurosurgery, Sanbo Brain Hospital, Capital Medical University, Beijing 100093, China; Department of Neurosurgery, Sanbo Brain Hospital, Capital Medical University, Beijing 100093, China; Nanjing Geneseq Technology Inc., Nanjing 211899, China; School of Public Health, Nanjing Medical University, Nanjing 211198, China; Department of Pathology, Sanbo Brain Hospital, Capital Medical University, Beijing 100093, China; Department of Pathology, Sanbo Brain Hospital, Capital Medical University, Beijing 100093, China; Department of Pathology, Sanbo Brain Hospital, Capital Medical University, Beijing 100093, China; Department of Radiology, Sanbo Brain Hospital, Capital Medical University, Beijing 100093, China; Department of Biology, Beijing Key Laboratory of Gene Resource and Molecular Development, School of Life Sciences, Beijing Normal University, Beijing 100875, China; Key Laboratory of Cell Proliferation and Regulation Biology, Ministry of Education, School of Life Sciences, Beijing Normal University, Beijing 100875, China; Department of Pathology, Sanbo Brain Hospital, Capital Medical University, Beijing 100093, China

**Keywords:** low-grade glioneuronal tumour, neural lineage, differentiation stage, oligodendrocyte precursor cells, RAS/MAPK pathway

## Abstract

Current histological classification of low-grade glioneuronal tumours does not adequately represent their underlying biology. The neural lineage(s) and differentiation stage(s) involved and the cell state(s) affected by the recurrent genomic alterations are unclear. Here, we describe dysregulated oligodendrocyte lineage developmental programmes in three low-grade glioneuronal tumour subtypes. Ten dysembryoplastic neuroepithelial tumours, four myxoid glioneuronal tumours and five rosette-forming glioneuronal tumours were collected. Besides a comprehensive characterization of clinical features, known diagnostic markers and genomic alterations, we used comprehensive immunohistochemical stainings to characterize activation of rat sarcoma/mitogen-activated protein kinase pathway, involvement of neuronal component, resemblance to glial lineages and differentiation blockage along the stages of oligodendrocyte lineage. The findings were further complemented by gene set enrichment analysis with transcriptome data of dysembryoplastic neuroepithelial tumours from the literature. Dysembryoplastic neuroepithelial tumours, myxoid glioneuronal tumours and rosette-forming glioneuronal tumours occur at different ages, with symptoms closely related to tumour location. Dysembryoplastic neuroepithelial tumours and myxoid glioneuronal tumours contain oligodendrocyte-like cells and neuronal component. Rosette-forming glioneuronal tumours contained regions of rosette-forming neurocytic and astrocytic features. Scattered neurons, identified by neuronal nuclei antigen and microtubule-associated protein-2 staining, were consistently observed in all dysembryoplastic neuroepithelial tumours and myxoid glioneuronal tumours examined, but only in one rosette-forming glioneuronal tumour. Pervasive neurofilament-positive axons were observed only in dysembryoplastic neuroepithelial tumour and myxoid glioneuronal tumour samples. Alterations in B-Raf proto-oncogene, serine/threonine kinase, fibroblast growth factor receptor 1, fibroblast growth factor receptor 3 and platelet-derived growth factor receptor alpha occurred in a mutually exclusive manner, coinciding with strong staining of phospho-p44/42 mitogen-activated protein kinase and low apoptotic signal. All dysembryoplastic neuroepithelial tumours, myxoid glioneuronal tumours and the neurocytic regions of rosette-forming glioneuronal tumours showed strong expression of neuron-glia antigen 2, platelet-derived growth factor receptor alpha (markers of oligodendrocyte precursor cells) and neurite outgrowth inhibitor-A (a marker of developing oligodendrocytes), but lacked the expression of oligodendrocyte markers ectonucleotide pyrophosphatase/phosphodiesterase family member 6 and myelin basic protein. Notably, transcriptomes of dysembryoplastic neuroepithelial tumours were enriched in oligodendrocyte precursor cell signature, but not in signatures of neural stem cells, myelinating oligodendrocytes and astrocytes. Dysembryoplastic neuroepithelial tumour, myxoid glioneuronal tumour and rosette-forming glioneuronal tumour resemble oligodendrocyte precursor cells, and their enrichment of oligodendrocyte precursor cell phenotypes is closely associated with the recurrent mutations in rat sarcoma/mitogen-activated protein kinase pathway.

## Teaser

Three low-grade glioneuronal tumour subtypes (dysembryoplastic neuroepithelial tumours, myxiod glioneuronal tumours and rosette-forming glioneuronal tumours) were examined for dysregulated oligodendrocyte lineage developmental programmes. They resembled oligodendrocyte precursor cells, and their blocked differentiation prior to the myelinating oligodendrocyte stage was closely associated with recurrent mutations in Ras/mitogen-activated protein kinase pathway.

## Graphical Abstract

**Graphical Abstract:**
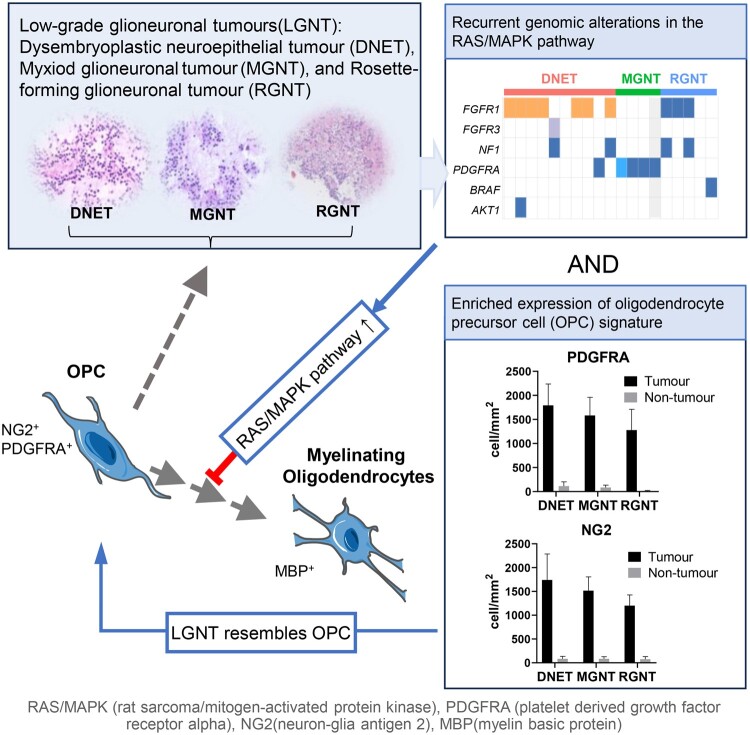

## Introduction

Low-grade glioneuronal tumours (LGNT) are a group of tumours containing both glial and neuronal components. A large fraction of LGNTs occur in the cerebral cortex and manifest early-onset epilepsy in children or young adults.^1^ LGNTs are hitherto diagnosed based on their histological features, localization and genomic alterations. Due to ambiguous and overlapping histopathological factures, LGNT classification has been challenging, and interobserver agreement across the subtypes is only 40%.^2^ While some recurrent genomic alterations are subtype-specific, other recurrent genomic alterations are overlapping across the subtypes.^1,3^ Though the majority of LGNTs are relatively benign and do not transform into high-grade tumour, subsets of LGNTs show dismal outcome,^4^ which cannot be explained by the histopathological features and genomic alterations. Dysembryoplastic neuroepithelial tumour (DNET) is the second most common type of LGNT.^5^ With its cortex location and multinodular architecture, DNETs exhibit a histological hallmark of pathogenic giloneuronal element, formed by oligodendrocyte-like tumour cells (OLCs) and floating entrapped cortical neurons.^5,6^ Alterations in fibroblast growth factor receptor 1 (FGFR1) (intragenic duplication, mutation or rearrangement) are frequent in DNET.^7,8^

Myxoid glioneuronal tumour (MGNT) is a newly proposed entity of LGNT,^1,9^ mostly located in the septal nuclei and septum pellucidum. MGNTs share similar morphological characteristics of DNETs, characterized by OLCs embedded in a mucoid matrix. However, multinodular growth pattern of cortical DNETs is not observed.^10,11^ MGNT was recently proposed as a new subtype; these tumours were considered as extracortical or septal DNET.^11^ Few cases may contain features of neurocytic rosettes, which is overlapping with rosette-forming glioneuronal tumours (RGNT).^10,12,13^ MGNTs rarely harbour astrocytoma component.^10,14^ In other words, MGNT can show histological features overlapping with DNET or RGNT. The characteristic genetic alteration of MGNT is the recurrent platelet-derived growth factor receptor alpha (PDGFRA): p.K385L/I mutation.^11^

RGNT is a rare, slowly growing type of tumour characterized by a biphasic neurocytic and glial architecture, which mainly located in or close to the fourth ventricle. The neurocytic element can be similar to that in DNET or MGNT.^15^ FGFR1 hotspot missense mutations within the tyrosine kinase domain (either at p.N546 or p.K656) were observed in almost all RGNTs reported so far, often with co-occurring mutations in PIK3CA, neurofibromatosis type 1 (NF1), or protein tyrosine phosphatase non-receptor type 11 (PTPN11).^16,17^

While current studies predominantly focus on genomic analysis and the related prognostic relevance,^18^ little is known about the neural lineage(s) involved and the interplay between potential cells of origin and those recurrent genomic alterations in LGNTs. Here, we collected a new cohort of LGNTs containing 10 cases of DNET, 4 cases of MGNT and 5 cases of RGNT with considerable overlap in morphology and genomic alterations. In addition to a comprehensive characterization of clinical and genomic features, we have taken a developmental approach to map the neural lineage and differentiation stage involved in these LGNTs. Our findings indicate that DNET, MGNT and RGNT share a common differentiation blockage in the early stages of oligodendrocyte lineage. Besides strong expression of pan-oligodendrocyte lineage markers oligodendrocyte lineage transcription factor 2 (OLIG2) and sex determining region Y-box 10 (SOX10), the tumour cells universally expressed PDGFRA and neuron-glia antigen 2 (NG2) [markers of oligodendrocyte precursor cells (OPC)^19^] and neurite outgrowth inhibitor-A (Nogo-A) (a marker of developing oligodendrocytes^20^), but lacked the expression of developing oligodendrocyte marker ectonucleotide pyrophosphatase/phosphodiesterase family member 6 (ENPP6) and mature oligodendrocyte maker myelin basic protein (MBP).^19^ The transcriptomes of DNETs showed enriched expression of OPC signature in comparison with the signatures of neural stem cells (NSC), myelinating oligodendrocytes (MO) and astrocytes (Astro). Thus, across the DNET, MGNT and RGNT tumours, recurrent genomic alterations may exert their effect on OPC-like cells with elevated rat sarcoma/mitogen-activated protein kinase (RAS/MAPK) pathway activity. Our findings may improve the diagnosis and raise alternative approaches facilitating pathogenic understanding and treatment development against LGNTs.

## Materials and methods

### Patient samples

Eighty-six DNET and 13 RGNT tumours treated at Sanbo Brain Hospital, Capital Medical University, between 2008 and 2021 were initially collected. These tumours were initially diagnosed based on histopathological, immunohistochemical and genomic characteristics of DNET or RGNT according to the 2016 WHO classification.^21^ According to the 2021 WHO classification,^1^ the septal DNETs were reclassified as MGNT based on the special location and PDGFRA: p.K385L/I mutation. All surgical pathology specimens were reviewed by two neuropathologists (Z.D. and X.Q.) who are co-authors of this report. Considering the availability of tissue specimen and representativeness in morphology and location, 10 classical cortical DNETs, 4 MGNTs located in the interventricular foramen and 5 RGNTs located in the supratentorial region were included in this study; the clinical data were retrieved through medical records (Table 1). This study was approved by the ethical committee of Sanbo Brain Hospital, Capital Medical University.

**Table 1:** Clinical features of DNET, MGNT and RGNT cases examined

Case ID	Gender	Age (years)	Tumour location	Presenting symptoms	Extent of resection	Adjuvant therapy	Recurrence or progression	Clinical outcome	Follow-up (months)
DNET-1	Female	13	Left occipital lobe	Epilepsy, intermittent dizziness, nausea, chest tightness with numbness of the upper limbs and unstable walking	Gross total	None	None	Alive without evidence of disease	36
DNET-2	Male	22	Left frontal lobe and corpus callosum	Epilepsy, tonic seizures	Gross total	None	None	Epilepsy with seizures	30
DNET-3	Male	7	Right frontal lobe	Epilepsy, uncontrollable twitching left limbs	Gross total	None	None	Alive without evidence of disease	30
DNET-4	Female	27	Right temporal lobe	Epilepsy, tonic seizures	Gross total	None	None	Alive without evidence of disease	18
DNET-5	Male	18	Right temporal lobe	Epilepsy, absence seizures	Gross total	None	None	Alive without evidence of disease	26
DNET-6	Female	7	Left frontal lobe	Epilepsy, absence seizures, uncontrollable twitching limbs	Gross total	None	None	Alive without evidence of disease	24
DNET-7	Male	13	Right parietal lobe	Epilepsy, tonic–clonic seizures	Gross total	None	None	Alive without evidence of disease	21
DNET-8	Female	19	Right frontal lobe	Epilepsy, tonic seizures	Gross total	None	None (after the second surgery)	Alive without evidence of disease	20 (after the second surgery)
DNET-9	Female	7	Left temporal lobe	Epilepsy, tonic seizures	Gross total	None	None	Alive without evidence of disease	10
DNET-10	Male	13	Left occipital lobe	Epilepsy, absence seizures	Gross total	None	None	Alive without evidence of disease	10
MGNT-1	Female	20	Interventricular foramen	Mental disorder	Gross total	None	None	Alive without evidence of disease	14
MGNT-2	Female	33	Interventricular foramen	Headaches	Gross total	None	None	Alive without evidence of disease	11
MGNT-3	Female	21	Interventricular foramen	Visual impairment	Gross total	None	None	Alive without evidence of disease	13
MGNT-4	Female	46	Interventricular foramen	Headaches	Gross total	None	None	Alive without evidence of disease	9
RGNT-1	Female	16	Lateral ventricle	Headache, NF1 mutation	Gross total (the second surgery)	None	None (after the second surgery)	Alive without evidence of disease	40 (after the second surgery)
RGNT-2	Male	34	Sellar region	Fatigue, headache and hyposexuality	Gross total	None	None	Alive without evidence of disease	51
RGNT-3	Female	26	Sellar region	Headache, nausea, vomiting and narrow vision	Gross subtotal	None	None	Alive without evidence of recurrence or progression	49
RGNT-4	Female	29	Basal ganglia	Headache, disorder of consciousness, nausea and vomiting	Biopsy	None	Na	Alive with headache	60
RGNT-5	Female	51	Midbrain tectal region	Sleepiness, walking instability, blurred vision, memory loss	Biopsy	None	NA	NA	NA

### Immunohistochemical staining

Representative formalin-fixed paraffin-embedded (FFPE) sections were deparaffinized and stained with haematoxylin and eosin (H&E) and immunohistochemistry (IHC) according to the manufacturer’s instructions of the respective reagents. The following antibodies were used: (i) Astro makers: GFAP (Dako, EP13, 1:150) and S-100 (Dako, 15E2E2 + 4C4.9, 1:4000); (ii) neuronal makers: neuronal nuclei antigen (NeuN) (Zeta, A60, 1:100), neurofilament (NF) (Covance Research Products, 2F11, 1:200), microtubule-associated protein-2 (MAP-2) (Abcam, AP18, 1:100), synaptophysin (SYN) (Neomarkers, EP158, 1:50) and postsynaptic density protein-95 (PSD95, Abcam, EPR23124-118, 1:100); (iii) pan-lineage or stage-specific markers of oligodendrocyte lineage:^19^ OLIG2 (Dako, EP112, 1:100), SOX10 (Abcam, EP268, 1:200), NG2 (Cell Signaling Technology, E3B3G, 1:300), PDGFRA (Abcam, polyclonal, 1:100), Nogo-A (Sigma, 1:1000), ENPP6 (Atlas antibodies, polyclonal, 1:50) and MBP (Epitomics, EP207, 1:200); (iv) diagnostic markers: (1) for diffuse glioma: IDH1-R132H (Dianova, H09, 1:60), ATRX (Sigma, HPA001906, 1:800), TP53 (Santa Cruz Biotechnology, BP53.12, 1:200), H3K27M (EMD Millipore, ABE419, 1:3000) and H3K27me3 (Cell Signaling Technology, C36B11, 1:300), (2) CD34 (Dako, QBEnd/10, 1:100) mainly for differential diagnosis of ganglioglioma, (3) Ki-67 (Origene, UMAB107,1:100) for evaluating proliferative activity of tumour cells and (4) anti-B-Raf proto-oncogene, serine/threonine kinase (BRAF) V600E antibody for detecting the BRAF V600E protein expression (VE1, IHC detection kit, Ventana Medical Systems);^22^ (v) reporter of RAS/MAPK pathway activation: p-Erk1/2 (Cell Signaling Technology, D13.14.4E, 1:400); and (vi) anti-cleaved caspase-3 (Cell Signaling, Asp175, 1:400) for detection of apoptotic cells. The staining was performed using Leica Bond automated staining processors. The anti-BRAF V600E (VE1) immunostaining was automated and conducted on a Benchmark Ultra stainer from Ventana/Roche, utilizing the proprietary antigen retrieval solution provided by the manufacturer.^22^ Ten diffuse hemispheric gliomas (DHGs), H3 G34-mutant (as known as G34R/V mutant high-grade gliomas), in adolescents and young adults were used as a positive control for PDGFRA staining and negative controls for the other oligodendrocyte lineage markers because these tumours resemble interneuron progenitors and lack oligodendroglial programmes.^23^ The white matter adjacent to these tumour, normal brain tissues from traumatic brain injuries and oligodendroglioma (Grades 2 and 3) were used as positive controls for oligodendrocyte lineage markers, including OLIG2, Nogo-A, EPNN6 and MBP. The Luxol fast blue (LFB) staining was performed for detecting myelin sheath structure. The stainings of immunohistochemical diagnostic markers (except Ki-67) were graded according to the percentages of positively stained tumour cells and whether the positive staining was in the nucleus, cytoplasm or cell membrane (0, no staining; 1+, <5%; 2+, 5–25%; 3+, 26–50%; 4+, 51–100%). These ranges are frequently used in reporting the staining results of diagnostic markers.^24,25^

### Morphometry and statistical analyses of cell numbers

Using the ImageJ programme, the numbers of PDGFRA-, NG2-, OLIG2-, SOX10-, Nogo-A-, NeuN- and Caspase3-positive cells in IHC staining were determined in at least four microscopic fields (0.119 mm^2^/field) in each sample. The non-tumour regions from the same slide were selected as the control. In the text and table, the mean numbers of cells/mm^2^ ± standard deviation (SD) are presented. The R version 4.0.5 was used for diagram presentation (R package ggplot2 v.3.4.2) and statistical analysis (predefined functions in R). *P*-values < 0.05, *P* < 0.01 and 0.001 were indicated with *, ** and ***, respectively.

### Targeted exome sequencing

Targeted exome sequencing was performed in 10 DNETs, 3 MGNTs (MGNT 1–3) and 5 RGNTs as previously described.^26^ Areas of the FFPE specimens enriched in tumour cells were marked by HE staining and isolated for DNA preparation using QIAamp DNA Mini Kit (Qiagen). The quality and concentration of DNA preparations were examined using spectrophotometer (Biophotometer Eppendorf, Germany). Hybridization-based target enrichment was carried out with GeneseeqOne™ pan-cancer gene panel (425-cancer-relevant genes, Geneseeq Technology Inc.) (Supplementary Table 1) and xGen Lockdown Hybridization and Wash Reagents Kit (Integrated DNA Technologies). The libraries were sequenced on the HiSeq 4000 platform (Illumina, San Diego, CA) with 2 × 150 bp pair-end reads. Sequencing data were demultiplexed by bcl2fastq (v2.19) and analysed using Trimmomatic.^27^ The genome analysis toolkit (GATK)^28^ was used to perform local realignments around indels and base quality reassurance. SNPs and indels were called using VarScan2^29^ and HaplotypeCaller UnifiedGenotyper in GATK, with mutant allele frequency (MAF) cut-off of 0.5% for tissue samples and a minimum of three unique mutant reads. Common variants were removed using the reference data from dbSNP and the 1000 Genome project.

For four MGNTs and five RGNTs, PDGFRA mutations were analysed by direct sequencing of PCR products from tumour DNA using the primers reported by Chiang *et al*.^11^:

Forward primer: 5′-E8-F GTATGATCCAGACCTTCAGTG-3′

Reverse primer: 5′-E8-R CATCTACAGAGCTAGCATTATCT-3′

### Gene set enrichment analysis

Transcriptome data were obtained from a publicly available paediatric low-grade glioma dataset GSE60898.^30^ The expression values were log_2_ transformed prior to gene set enrichment analysis (GSEA) analyses. GSEA was performed with the GSEA^31^ software (https://www.gsea-msigdb.org/gsea/index.jsp) with default parameters. The signal2noise was used as the ranking metric. Single-sample GSEA (ssGSEA) was performed in R with GSVA R package. The signatures of different cell types were obtained from previous reports. The signature of NSCs represented the genes with characteristic expression profile in the adult human neurogenic subventricular zone Astro, the module M13C in Oldham *et al*.^32^ Stage-specific signatures of oligodendrocyte lineage [OPC, newly formed oligodendrocytes (NFO), MO] were a combination of the signature genes reported by Goldman *et al*.^19^ and the top 40 genes and the top 10 transcription factors reported by Zhang *et al*.^33^ The astrocytic signature genes included the top 40 genes and the top 10 transcription factors reported by Zhang *et al*.^33^ The gene lists of each signature, composed of the signature genes actually detected in GSE60898, are shown in Supplementary Table 4.

## Results

### Clinical features

The median age (at the initial surgery) of the 10 patients with cortical DNET was 13 years (range: 7–27 years), with a male-to-female ratio of 1:1. All DNET cases showed symptoms of epilepsy. The duration of symptoms ranged from 10 days to several years. All four MGNT patients were female with a median age of 27 years (range: 21–46 years). The presenting symptoms included headache (two cases), mental disorder (one case) and visual impairment (one case). None of four MGNT cases showed symptoms of epilepsy. The median age of the four female patients and one male patient with RGNT was 29 years (range: 16–34 years) (Table 1). The symptoms for the RGNT patients included headache (four cases), nausea and vomiting (two cases). RGNT-1 was a secondary tumour with NF1 mutation, whose first diagnosis was haemangioma 6 years ago, and all other RGNTs were primary cases. All 10 DNETs, 4 MGNTs and 3 of the 5 RGNTs tumours were resected totally with craniotomy resection or ventriculoscope operation; the remaining two RGNTs were treated with fistula operation and biopsy. All patients did not receive adjuvant therapy after surgery. There was no evidence of recurrence or progression by MRI examination during the follow-up period, which ranged from 9 to 60 months (median: 22.5 months).

### Imaging features

**Figure 1:**
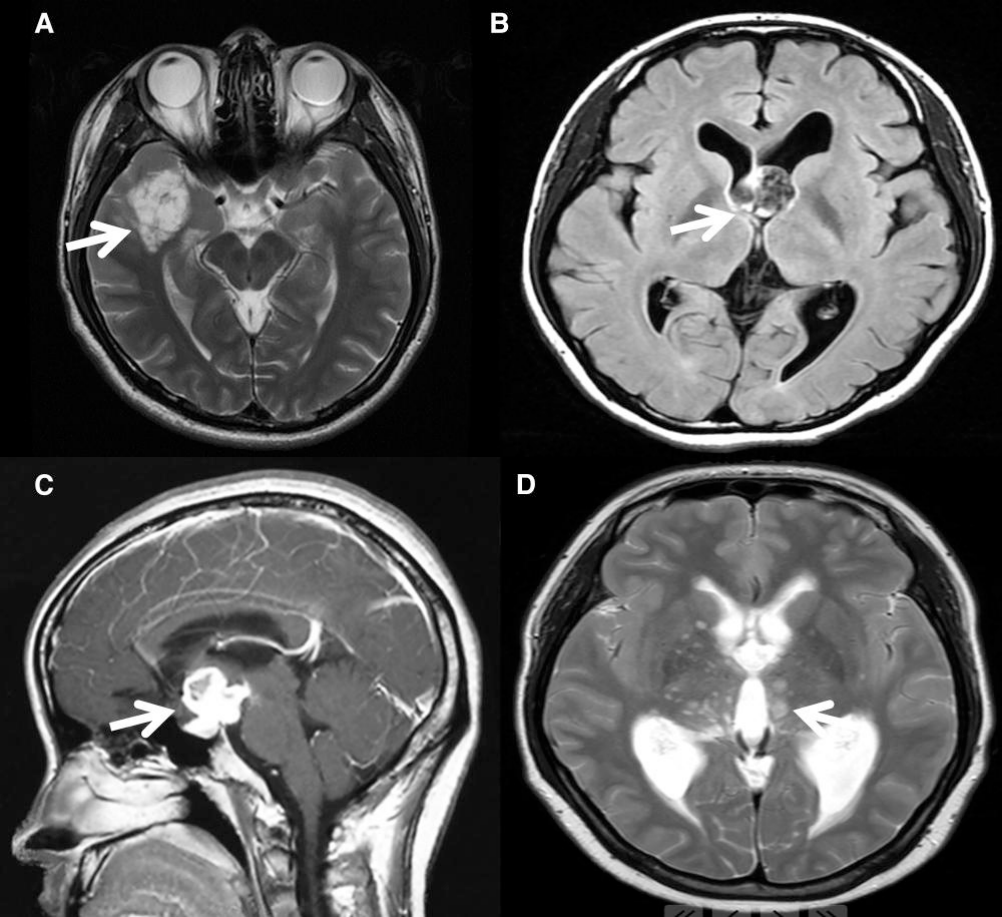
**Preoperative images of representative cases in this study.** (**A**) Preoperative MRI image of DNET-5 showing a hyperintensity on T_2_-weighted images of thickening cortex within temporal lobe without surrounding tumoural oedema. (**B**) Preoperative MRI image of MGNT-2 showing a well-circumscribed, FLAIR isointensity or hyperintense mass lesion in the interventricular foramen. (**C**) Preoperative MRI image of RGNT-3 showing a lump mass located in the sellar region with abnormal enhancement. (**D**) Multiple circular signals in the bilateral basal ganglia and medial temporal lobes were observed in RGNT-4. Arrows indicate the lesion.

**Cortical DNETs:** preoperative MRI showed irregular or round flaky abnormal signal within thickening cortex, mainly involving temporal lobe and frontal lobe. The lesions were hypointense on T_1_-weighted images (T1WI) and hyperintense on T_2_-weighted images (T2WI), without obvious contrast enhancement (Fig. 1A). The surrounding tumoural oedema was not obvious.

**MGNTs:** preoperative MRI showed that lesions were in the interventricular foramen with clear circular boundary. All lesions showed hypointensity on T1WI, hyperintensity on T2WI and isointensity/hyperintensity on fluid-attenuated inversion recovery (FLAIR) without contrast enhancement (Fig. 1B). Obstructive hydrocephalus was present in all four cases. Based on the imaging data, preoperative diagnoses included subependymoma, neuroepithelial cyst and endodermal cyst.

**RGNTs:** preoperative MRI showed that lesions were located outside the fourth ventricle, including lateral ventricle, sellar region, basal ganglia and midbrain tectal region. The lesions were hypointense on T1WI and hyperintense on T2WI, Cases 1 and 3 showed enhancement (Fig. 1C and D). Ventricular dissemination was not detected at the time of diagnosis for all MGNT and RGNT cases in this study.

### Morphological features and expression of glial and diagnostic markers in LGNTs

**Figure 2:**
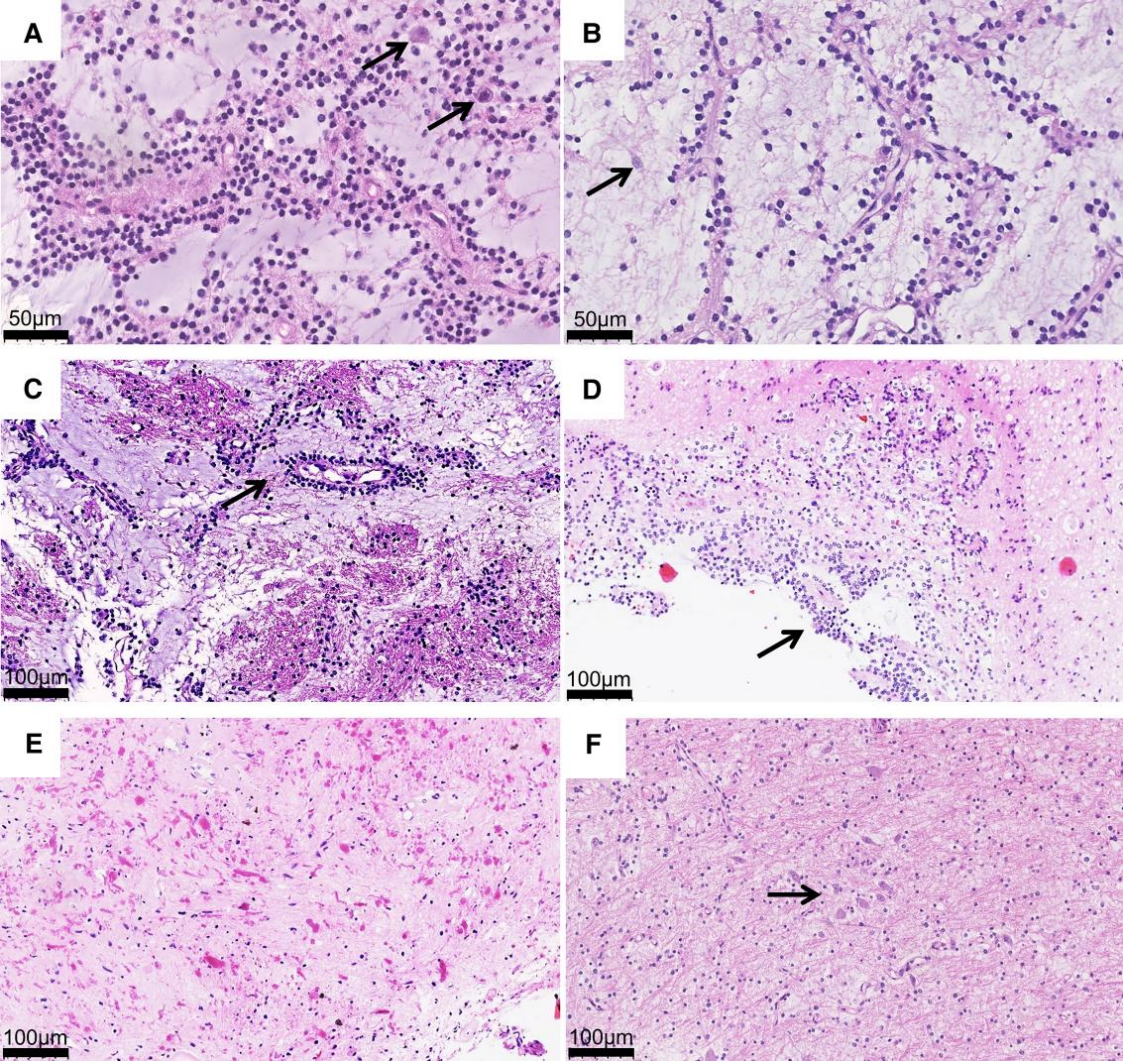
**Morphological features of DNET, MGNT and RGNT samples examined.** HE stainings of representative DNET case (**A**) and MGNT case (**B**). Tumours contained OLCs with small monotonous round to oval nuclei. Proliferative cells were rare. Scattered neurons were found in the mucinous matrix (black arrows) (×400). (**C**) Tumour tissue was infiltrating into the white matter in the form of push invasion in MGNT-2 (black arrow) (×200). Typical RGNT (RGNT-2) contained a rosette-forming neurocytic component (black arrow indicating neurocytic rosettes and perivascular pseudorosettes) (**D**) and an astrocytic component (**E**) (×200). (**F**) MGNT-1 contained regions with scattered and clustered neurons, which is similar to normal white matter (×200).

DNETs and MGNTs shared highly similar histological characteristics (Fig. 2A and B and Supplementary Fig. 1). These tumours contained OLCs with small monotonous round to oval nuclei, scant to moderate eosinophilic or transparent cytoplasm. All samples contained a prominent mucoid matrix. Mitotic figures were rare or not observed. Neither necrosis nor glomeruloid microvascular proliferation was detected. However, scattered proliferative Astro were identified. Calcifications, rosenthal fibres and eosinophilic granular bodies were not observed. Except for DNET-2, the multinodular intracortical growth pattern was observed in other nine DNET samples examined. However, multinodular architecture was not observed in MGNTs. In two MGNT cases, we observed tumour infiltration into the adjacent white matter in the form of pushing invasion (Fig. 2C). All five RGNT cases showed common morphological features with two distinct regions characterized by a rosette-forming neurocytic component (Fig. 2D and Supplementary Fig. 1) and an astrocytic component (Fig. 2E). The rosette-forming neurocytic component contained neurocytic rosettes and perivascular pseudorosettes, which were embedded in a mucoid matrix in RGNT-1 to RGNT-4; no obvious mucoid matrix was observed in RGNT-5. The astrocytic component was similar to that in pilocytic astrocytoma. Some rosenthal fibres and eosinophilic granular bodies were observed in the astrocytic regions in RGNT-1 to RGNT-3.

The DNET, MGNT and RGNT samples examined presented similar immunohistochemical features (Supplementary Table 2). Staining of Astro marker S-100 was observed in all cases, while GFAP expression was positive only in the astrocytic component of RGNTs and focally positive in three DNETs (DNET-4, -5 and -7). MAP-2 staining was diffusely positive in OLCs in all samples, but its expression was weaker in DNET samples than in MGNT and RGNT samples. Granular SYN staining was observed throughout the background neuropil in all three types of LGNT and especially at the centre of neurocytic rosettes and perivascular pseudorosettes in the RGNT samples (Supplementary Fig. 2). Stainings for IDH1-R132H, BRAF V600E, P53 and H3K27M were negative in all cases. None of cases showed loss of H3K27me3 expression. Except for diffuse staining in RGNT-5 and focal positive staining in DNET-5 and DNET-8, CD34 immunoreactivity was not observed in all other cases. The Ki-67 labelling index was low, ranging from 1% to 4%, and predominantly in the astrocytic region in RGNT samples, indicating most of the LGNTs were not proliferating.

These observations show that our cohort is consistent with previous findings on the overlapping morphological and immunohistochemical features between the LGNT subtypes.^1,2^

### Mutually exclusive genomic alterations and elevated RAS/MAPK activity in LGNTs examined

**Figure 3:**
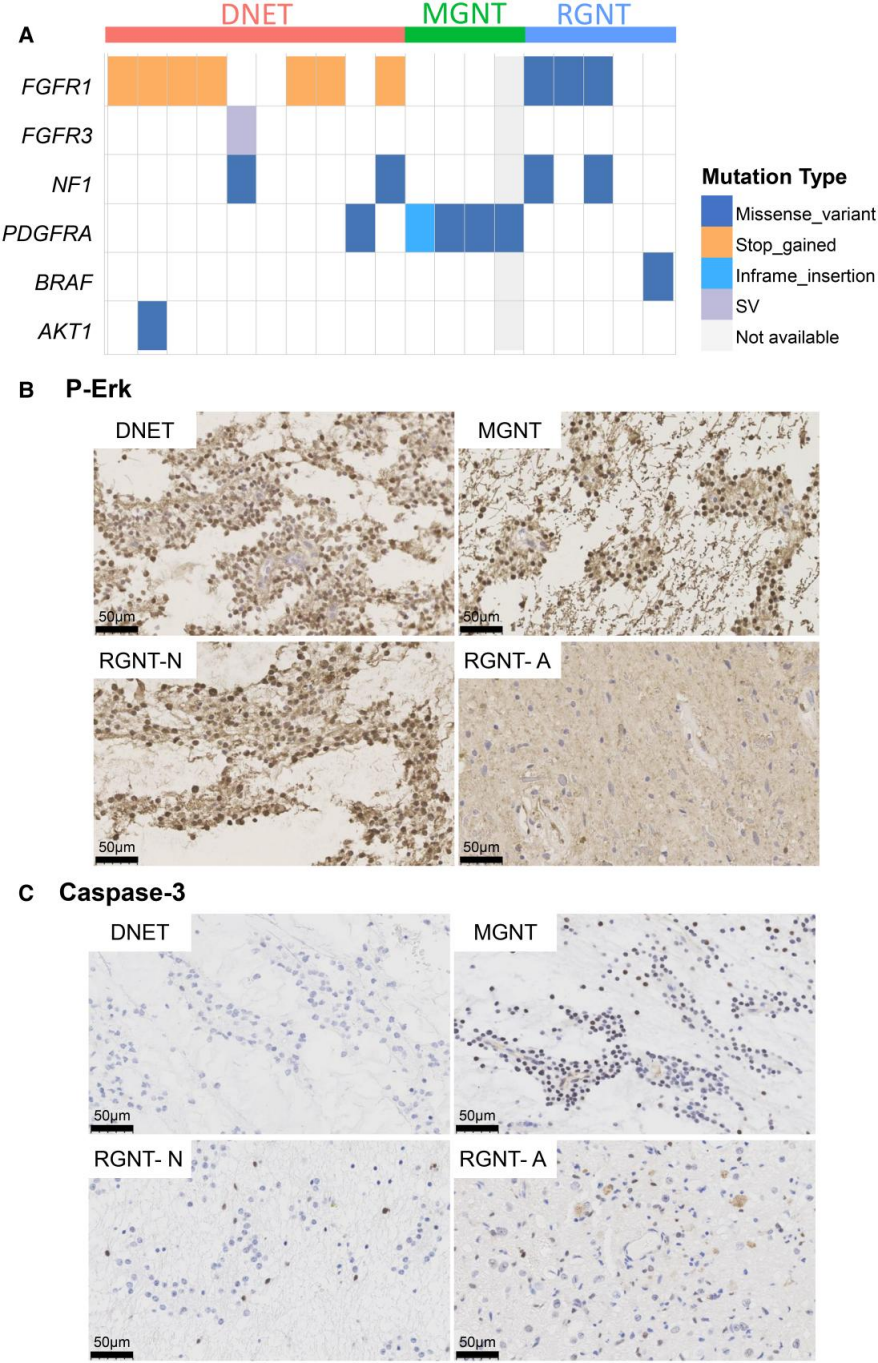
**Mutually exclusive genomic alterations leading to elevated RAS/MAPK and low apoptotic activities in DNET, MGNT and RGNT tumours.** (**A**) Recurrent genomic alterations in the RAS/MAPK pathway. In 19 LGNTs analysed, 17 LGNTs harboured 1 or 2 alterations along the RAS/MAPK pathway. Except for mutations in NF1, alterations in BRAF and receptor tyrosine kinases occurred in a mutually exclusive manner across the samples. AKT1, v-akt murine thymoma viral oncogene homologue 1. (**B**) Representative immunohistochemical images of p-Erk1/2 staining in DNET, MGNT and RGNT samples. Strong p-Erk1/2 staining was observed in all DNET and MGNT samples examined and in the neurocytic components of RGNTs (×400, scale bar: 50 *μ*m). (**C**) Anti-cleaved caspase-3 staining in DNET, MGNT and RGNT samples (×400, scale bar: 50 *μ*m).

To characterize genomic alterations in our cohort, we performed targeted exome sequencing for 425 cancer-related genes and Sanger sequencing for validation of PDGFRA mutation. Alterations in the drivers commonly affected in the other brain tumours, including IDH1/2, TP53, ATRX, TERT, EGFR and QKI, were not detected.^1^ Across the LGNT samples, mutual exclusivity was observed between the recurrent genomic alterations in BRAF, FGFR1, FGFR3 and PDGFRA (Fig. 3A). In the 10 cortical DNET examined, 7 harboured different mutations in FGFR1 (6 in exon 18 and 1 in exon 14, with MAF ranging 7.3–31.0%, mean 19.6%), 1 harboured FGFR3 structure variation (SV) (exon17∼FAM184B:exon2, MAF at 34.5%), and another 1 harboured PDGFRA p.K385L mutation with MAF at 21.7%. The remaining one DNET sample harboured BRCA2 mutation (c.10255dup, p.*3419Lfs*19, MAF at 43.17%), with no alterations detected along the RAS/MAPK pathway. All four MGNT cases harboured PDGFRA mutation. Among them, MGNT-2, -3 and -4 carried the typical c.1153-1154AA>TT, p.K385L PDGFRA mutation^10,11,12^ with MAF at 38.2% and 44.4% in MGNT-2 and MGNT-3, respectively. The MAF result of MGNT-4 was not available because this sample was only analysed using Sanger sequencing. MGNT-1 harboured an in-frame insertional mutation in PDGFRA (p.E362-I363insW) with MAF at 25.8%, a new variant not reported before in MGNTs. Three of the five RGNTs harboured FGFR1 kinase domain hotspot missense mutation, p.N546K in exon 12 with MAF at 27.3% in RGNT-1, p.N546D and p.K656Q with the respective MAF at 25.7% and 24.7% in exon 12 and exon 14 in RGNT-2 and p.K656E in exon 14 with MAF at 66.7% in RGNT-3.^17^ Among them, two harboured NF1 mutation and one harboured CDKN2B mutation. No genetic alteration along the RAS/MAPK pathway was detected in the RGNT-4. BRAF^V600E^ mutation was found in RGNT-5 with MAF at 23.4%. No copy number variations were detected in these cases.

To characterize the signalling effects of these genomic alterations, we performed staining for p-Erk1/2, a marker of RAS/MAPK pathway activity. Strong and diffuse nuclear staining of p-Erk1/2 was observed in all cases of DNET and MGNT tumours analysed. In the four RGNT-like tumours analysed (except RGNT-4 due to lack of material), neurocytic regions showed strong and diffuse p-Erk1/2 staining, though p-Erk1/2 staining was relatively weaker and less in astrocytic regions compared with neurocytic regions (Fig. 3B and Supplementary Fig. 3). Interestingly, strong p-Erk1/2 staining was also detected in two samples (DNET-6 and RGNT-4) with no detectable alterations in our panel.

ERK1/2 activation is broadly associated with anti-apoptotic function and thereby promotes cell survival.^34,35^ To evaluate the apoptotic level of these tumours, we performed anti-cleaved caspase-3 staining. The proportion of tumour cells with cleaved caspase-3 was 3.30 ± 1.25% in DNET samples (*n* = 10), 3.00 ± 1.41% in MGNT samples (*n* = 4) and 3.20 ± 3.90% and 6.00 ± 4.18% in the neurocytic and astrocytic regions of RGNT samples (*n* = 4), respectively (Fig. 3C and Supplementary Fig. 4). These results indicate a low level of apoptotic death of tumour cells in these LGNT samples.

Together, the mutual exclusivity between the mutations along the RAS/MAPK pathway and their relatively high MAF reinforce the conclusion that the majority of the LGNTs are driven by a single genetic alteration, which stands a good chance to result in elevated RAS/MAPK pathway activity and enhanced cell survival.^2,36^

### Pervasive axon components in the DNET and MGNT samples examined

Scattered neurons were found in the mucinous matrix in all DNET and MGNT samples analysed by H&E staining (black arrows in Fig. 2A and B, Supplementary Table 2). Interestingly, the pattern of neuron scattering in several regions in MGNT-1 and MGNT-2 was similar to that in normal white matter (Fig. 2F). However, no distinct neurons were observed in the RGNT samples (Fig. 2D and E).

**Figure 4:**
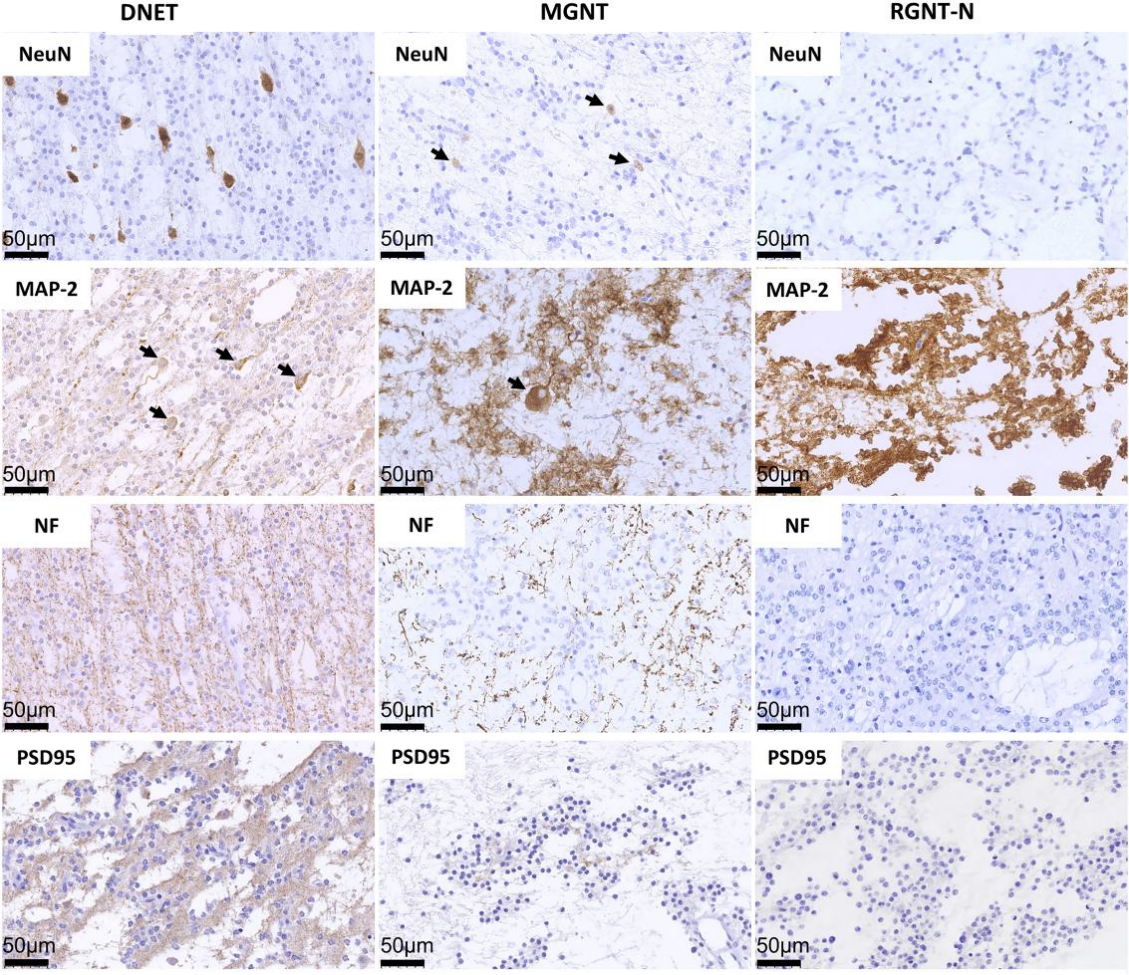
**Pervasive axon components in DNET and MGNT samples examined.** Representative IHC images of NeuN, MAP-2, NF and PSD95 stainings in DNET, MGNT and RGNT samples are shown (×400). In DNET samples, NeuN- and MAP-2-stained neuronal cell bodies were scattered in the tumour mass, while NF- and PSD95-stained axons were widely dispersed. In MGNT samples, NeuN and MAP-2 staining showed the rare neurons, while NF- and PSD95-stained axons were less widely dispersed. However, NeuN, MAP-2, NF and PSD95 stainings were negative in almost all RGNT samples examined. Scale bar: 50 *μ*m.

We performed IHC staining to confirm the distribution of neurons. NeuN staining can be used to indicate perikarya and proximal neuronal processes in nearly all neuronal populations.^37^ MAP-2 staining indicates neuron cell body and dendritic branching,^38^ NF staining indicates the axon structure,^39^ and PSD95 is a key postsynaptic protein located preferentially in dendritic spines, as its staining concentrates in the cytoplasm and neurites of neurons.^40,41^ In all DNET and MGNT samples analysed, sporadically dispersed neurons, positively stained with NeuN and MAP-2, were observed between the OLC-like cells (Fig. 4, Supplementary Figs. 5 and 6 and Supplementary Table 3). Compared with the DNET samples, less neuronal component was observed in the MGNT samples. However, except for RGNT-1 containing rarely dispersed NeuN-positive neurons, clear and distinct neuronal component was not observed in the other four RGNT samples analysed (Fig. 4). Axon structures were nearly homogeneously spread within the tumour tissues of DNET, but to a less extent or absent in the tumour tissues of MGNT and RGNT (Fig. 4 and Supplementary Fig. 5). Focal positive NF staining was observed in the neurocytic component of RGNT tumours (Fig. 4). In the traumatic brain samples and normal brain regions from tumour samples, PSD95 staining was confined to the cortex. PSD95 staining was weakly positive in DNET samples, further weaker in MGNT and almost absent in RGNT samples (Fig. 4 and Supplementary Fig. 5). SYN, an integral membrane glycoprotein and previously considered specific marker for neuronal lineage,^42,43^ showed diffuse positive staining in the background neuropil in all samples examined (Supplementary Fig. 2). Thus, SYN staining is unlikely associated with neuronal component in LGNTs examined. Together, these findings led us to speculate that homogeneously spreading axons may constitute a microenvironment niche towards the OLC-like cells in the DNET and MGNT samples.

### **DNET, MGNT and RGNT** tumour**s resemble early stages of oligodendrocyte lineage**

Mapping LGNT tumours to the neural lineages and differentiation stages may identify the neural cell state(s) affected by the recurrent genomic alterations.^2^ Using immunohistochemical staining for the markers of the consequential stages in oligodendrocyte lineage in our FFPE materials and GSEA analysis for the signatures of glial lineages/differentiation stages in transcriptomic data of DNET samples from the literature,^30^ we aimed to characterize the resemblance of LGNTs to glial lineages and stages. Based on the available antibodies and FFPE materials, we performed immunohistochemical staining to assess the expression of OLIG2 and SOX10 as markers of pan-oligodendrocyte lineage,^19^ NG2 and PDGFRA as markers of OPCs,^19^ Nogo-A as a marker of developing oligodendrocytes,^20,44^ ENPP6 as a marker of NFO^45,46^ and MBP as a marker of mature oligodendrocytes.^19^

**Figure 5:**
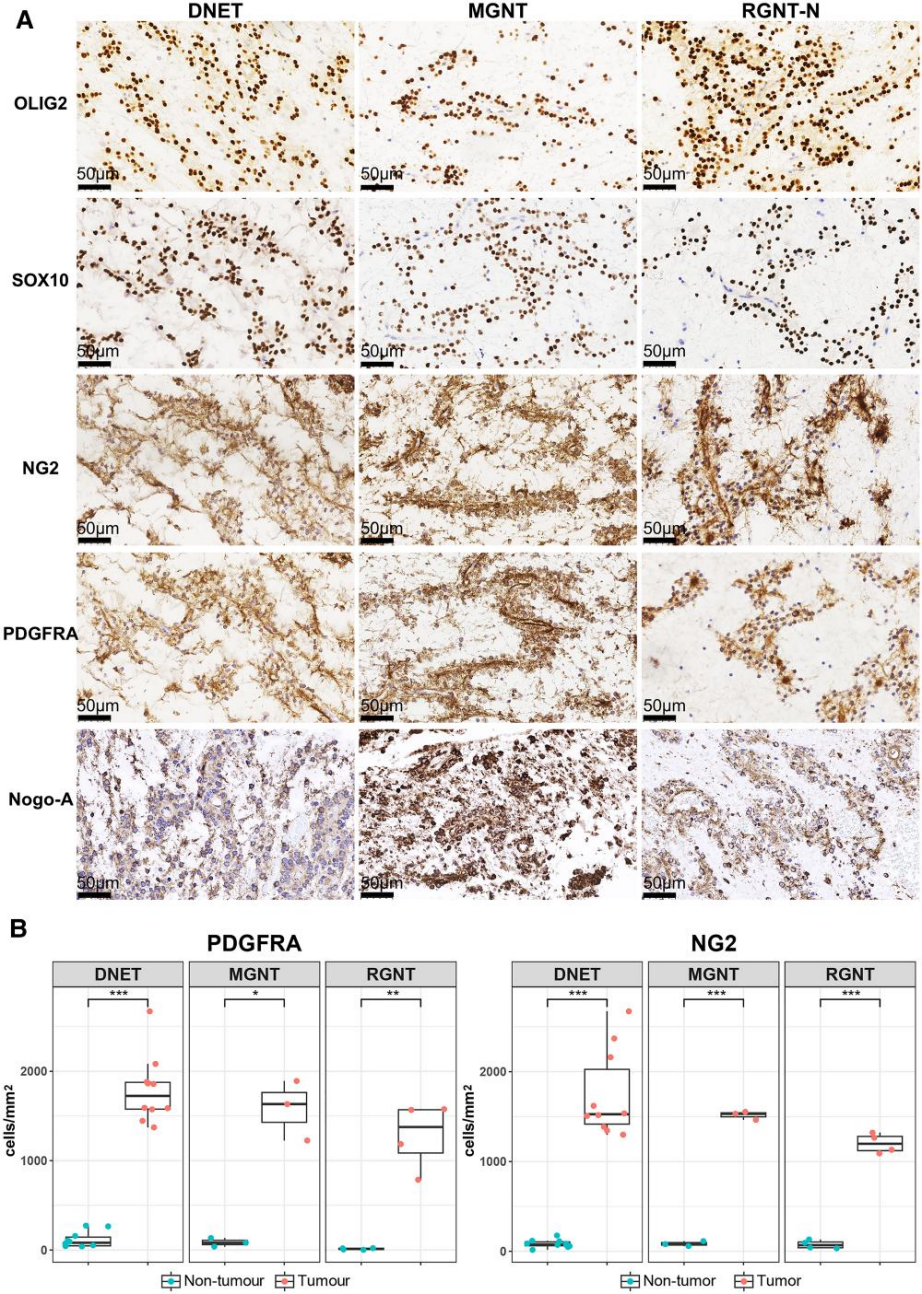
**Concordant expression of pan-oligodendrocyte lineage markers and OPC markers and dominating cell population expressing OPC markers in DNET, MGNT and RGNT samples analysed.** (**A**) IHC images of representative DNET (DNET-2), MGNT (MGNT-1) and the neurocytic (RGNT-N) regions of RGNT (RGNT-1) cases are shown. Strong positive stainings of OLIG2, SOX10, NG2, PDGFRA and Nogo-A in DNET, MGNT and RGNT-N tumour cells were observed. Images were taken at ×400. Scale bar: 50 *μ*m. (**B**) Quantification revealed significant increase in cells expressing PDGFRA and NG2. (Left) PDGFRA: DNET, *N* = 10, ***: paired *t*-test, *t* = −16.184, *P* = 5.821*e*^−08^; MGNT, *N* = 3, *: paired *t*-test, *t* = −7.0336, *P* = 0.01962; RGNT, *N* = 4, **: paired *t*-test, *t* = −6.8238, *P* = 0.006439; (Right) NG2: DNET, *N* = 10, ***: paired *t*-test, *t* = −10.94, *P* = 1.686*e*^−06^; MGNT, *N* = 3, ***: paired *t*-test, *t* = −69.412, *P* = 0.0002075; RGNT, *N* = 4, ***: paired *t*-test, *t* = −17.691, *P* = 0.0003938. Each data point represents the average number of positive cells/mm^2^ in an individual sample. Detailed data was included in Supplementary Table 3.

In the 10 DNET and 4 MGNT samples examined, OLIG2, SOX10, NG2 and PDGFRA were concordantly expressed; compared with the normal regions, tumour regions contained markedly more cells concordantly stained with OPC markers NG2 and PDGFRA, as well as cells positively stained with the pan-oligodendrocyte lineage marker OLIG2 and SOX10 (Fig. 5, Supplementary Figs. 6–8 and Supplementary Table 3). DNETs often exhibit multinodular growth pattern; the same staining patterns of OLIG2, SOX10, NG2 and PDGFRA were observed between the nodules within each DNET sample (Supplementary Fig. 7). In the RGNT samples, compared with the non-tumour regions, strong stainings of OLIG2, SOX10, NG2 and PDGFRA were observed in the neurocytic regions in all samples analysed, and the astrocytic regions of the same samples showed sporadic and weaker expression of OLIG2, SOX10, NG2 and PDGFRA (Fig. 5 and Supplementary Fig. 6).

Diffuse cytoplasmic staining of Nogo-A was observed in OLCs in all samples (Fig. 5, Supplementary Figs. 6 and 8 and Supplementary Table 3); positive staining of Nogo-A was also observed in astrocytic component of RGNT, white matter oligodendrocytes, parts of neurons and the background myelin sheaths.^47,48^ Tumour cells showed negative staining for ENPP6 and MBP in all samples analysed (Supplementary Fig. 9). The tumour regions also lacked the myelin structure as assessed with LFB staining (Supplementary Fig. 9). Stainings of ENPP6, MBP and LFB were largely negative in both types of regions in all five RGNT samples examined (Supplementary Fig. 9). As control, stainings of ENPP6, MBP and LFB were observed in the non-tumour regions.

To further assess the specificity of our staining, we performed the same set of stainings in 10 DHG samples, which resemble interneuron progenitors but lack oligodendroglial programme.^23^ Though positive staining of PDGFRA was observed in all cases, OLIG2 staining was not observed in all cases and scattered positive stainings of NG2 and SOX10 were observed in four cases and one case, respectively (Supplementary Fig. 10).

**Figure 6:**
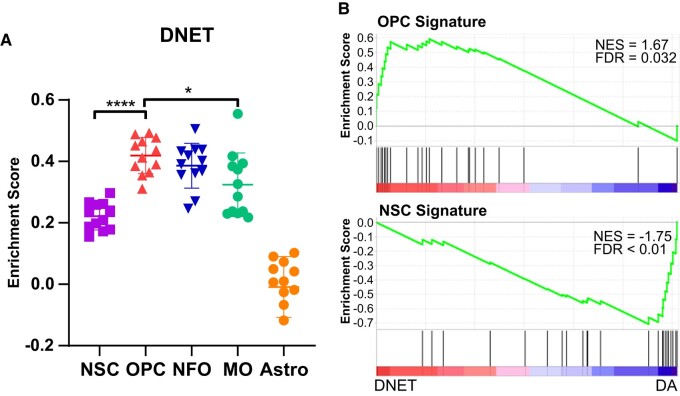
**Enrichment of OPC signature in DNET tumours.** (**A**) Single-sample GSEA with signature genes of NES, OPC, NFO, MO and Astro in DNET. Each data point represents enrichment score of an individual sample. The middle thick line represents the median enrichment score; the lower and upper limits represent standard deviation of the enrichment score. Adjusted *P*-values for ANOVA test with *post hoc* Bonferroni correction are indicated as *P < 0.05 and ****P < 0.0001. (**B**) Preranked GSEA with the OPC or NSC signature in DNET transcriptomes versus DA transcriptomes. NES, normalized enrichment score; FDR, false discovery rate.

To substantiate the above findings, we used single-sample GSEA (ssGSEA) to assess whether the signatures of NSC,^32^ OPCs, NFO, MO and Astro^19,33^ (Supplementary Table 4) are coordinately up- or downregulated in the DNET transcriptomes from the 12 individual samples in GSE60898.^30^ OPC signature was the most enriched signature in the DNET transcriptomes, followed by the signatures of NFO, MO^19,33^ and NSC;^32^ compared with the enriched OPC signature, enrichment of MO signature was significantly declined; the signature of Astro was not enriched in the DNET transcriptome signature (Fig. 6A). Compared with the 10 diffuse astrocytomas (DA), which were related to NSC with high expression of NSC characteristic genes,^49-51^ the 12 DNETs showed enriched expression of OPC signature instead of NSC signature (Fig. 6B). Similar analysis was not performed for MGNT and RGNT due to lack of the transcriptome data. In summary, our findings indicate that DNET, MGNT and RGNT tumours resemble early stages of oligodendrocyte lineage.

## Discussion

We have collected a new cohort of LGNT samples and performed an integrated analysis of clinical, genomic and neural developmental features. Besides reinforcing previous observations of genomic alterations in RAS/MAPK pathway as the drivers of LGNT, our study focused on the analyses of the glial lineage(s) and differentiation stage(s) involved in the subset of LGNTs examined. Though expression of individual markers of Astro (S-100 and GFAP) was detected in immunohistochemical staining, astrocytic gene signature was not enriched in the transcriptomes of DNETs. In contrast, our findings show nearly universal staining of pan-oligodendrocyte lineage markers OLIG2 and SOX10, OPC makers PDGFRA and NG2 and developing oligodendrocyte marker Nogo-A in the tumour cells of DNET, MGNT and RGNT samples and an enrichment of OPC signature in DNET transcriptomes. However, enrichment of MO signature was markedly reduced, markers of NFO (ENPP6) and mature oligodendrocyte (MFB), and myelin fibres were not detected in the tumour regions of our cohort. The LNGTs examined here may thus resemble early stages of oligodendrocyte lineage. The recurrent genomic alterations may affect OPC-like malignant cells with elevated RAS/MAPK activity. In analogue to OPC development,^52,53^ the widespread neuronal component appears to provide an interactive microenvironment to the OPC-like malignant cells in LGNT tumours. Together, our findings suggest that subsets of LGNTs share a common developmental dysregulation in oligodendrocyte lineage driven by uncontrolled RAS/MAPK activity.

LGNTs are currently diagnosed based on morphological features in combination with their locations and genomic alterations. This diagnostic scheme does not adequately represent their underlying biology. Due to ambiguous morphological features, a substantial fraction of LGNTs cannot be assigned into certain histological subtype. Though it is well known that LGNTs contain both glial and neuronal components,^1^ the underlying mechanism of their co-existence has been inadequately characterized. Our findings of the nearly universal expression of OPC markers PDGFRA and NG2, the enrichment in OPC signature and gradual decline in the enrichment scores of NFO and MO signature and the lack of expression of mature oligodendrocyte marker MFB and myelin structure suggest that LGNTs assessed in our cohort resemble OPCs.

**Figure 7:**
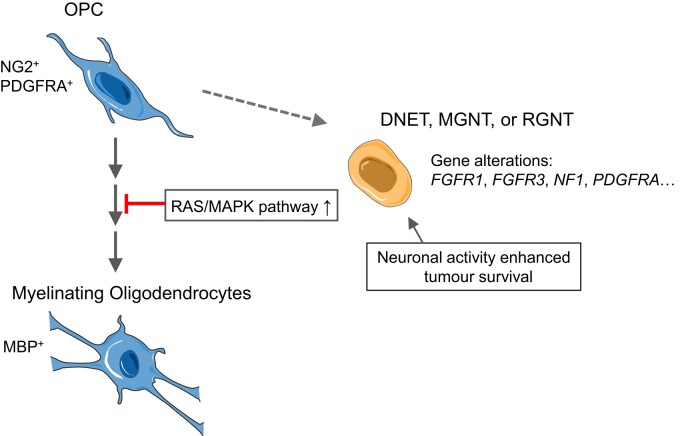
**A schematic model depicting the interplay between the neural cell states and driving genomic alterations in the LGNTs examined.** LGNTs are currently diagnosed based on histological features; biologically distinct entities among LGNTs have been thus far unclear. Characteristics of the neural cell states involved may provide a consensus for LGNT diagnosis that converges the features of glial lineage/differentiation stage involved and the driving signalling pathway and thereby defines biologically distinct entities in LGNTs.

Along the path of OPC differentiation into postmitotic, pre-MO and MO, early oligodendrocyte lineage cells are supported by the neuronal activities for proliferation and differentiation.^52,53^ The scattered neuronal cell body (NeuN staining) and the pervasive spread of axons (NF staining) and dendrites (MAP-2 staining) suggest that in analogy to OPC development, tumour cells in at least subsets of LGNTs may also be supported by neuronal activities, which may constitute a microenvironment niche involved in the maintenance of OPC-like malignant cells. Our findings of an enrichment of OPC phenotypes may provide an alternative model to integrate LGNT diagnosis with their neural cell states and recurrent mutations in the RAS/MAPK pathway (Fig. 7).

Using unbiased approach, a recent study reports two transcriptomic subtypes of LGNTs, which are enriched in astrocytic and oligodendroglial features, respectively.^54^ We believe that the discrepancy between this report and our findings is most likely caused by the fact that bulk expression profiles reflect the sum of all cell types within a tumour sample. As reported in the studies of molecular classification for adult gliomas, transcriptomic subtypes tend to be influenced by the proportion of non-malignant cells within glioma samples.^55,56^

Our findings confirm previous reports that most LGNTs harbour recurrent, but solitary, genomic alterations in members of the RAS/MAPK pathway. Further, these alterations occur in a mutually exclusive manner with relatively high MAF, indicating that genomic alterations in the RAS/MAPK pathway are initiating or early driving events in LGNT genesis. While the cell states affected by these genomic alterations were unclear in previous studies,^7,8,10-12,16-18^ our findings suggest that the resultant uncontrolled RAS/MAPK pathway activity may exert their effects in OPC-like malignant cells. Though our findings mirror early works on blocked OPC differentiation due to elevated FGF2-FGFR1 signalling,^57^ whether elevated RAS/MAPK pathway activity causes differentiation blockage or overall cell death and whether neuronal components support the OPC-like cells in DNET, MGNT and RGNT remain to be assessed in animal model experiments.

Our studies are limited because the cohort size was small; the findings thus require further validation in additional cohorts. Despite these limitations, our findings of a common enrichment of OPC phenotypes in MGNT, RGNT and DNET tumours may expedite the improvement of LGNT diagnosis. Future characterization of neuronal activity and evaluated RAS/MAPK activities exerted on OPC-like malignant cells may provide an alternative perspective for treatment development against LGNTs and other low-grade gliomas.

## Supplementary Material

fcae156_Supplementary_Data

## Data Availability

The data supporting the findings of this study are available on request from the corresponding author, X.Q.
