# Supplementary material for: Enrichment of oligodendrocyte precursor phenotypes in subsets of low-grade glioneuronal tumours
Source: Brain Commun. 2024 May 6;6(3):fcae156. doi: 10.1093/braincomms/fcae156 (PMC11099663; doi:10.1093/braincomms/fcae156)
Supplement: fcae156_Supplementary_Data [file fcae156_supplementary_data.pdf]

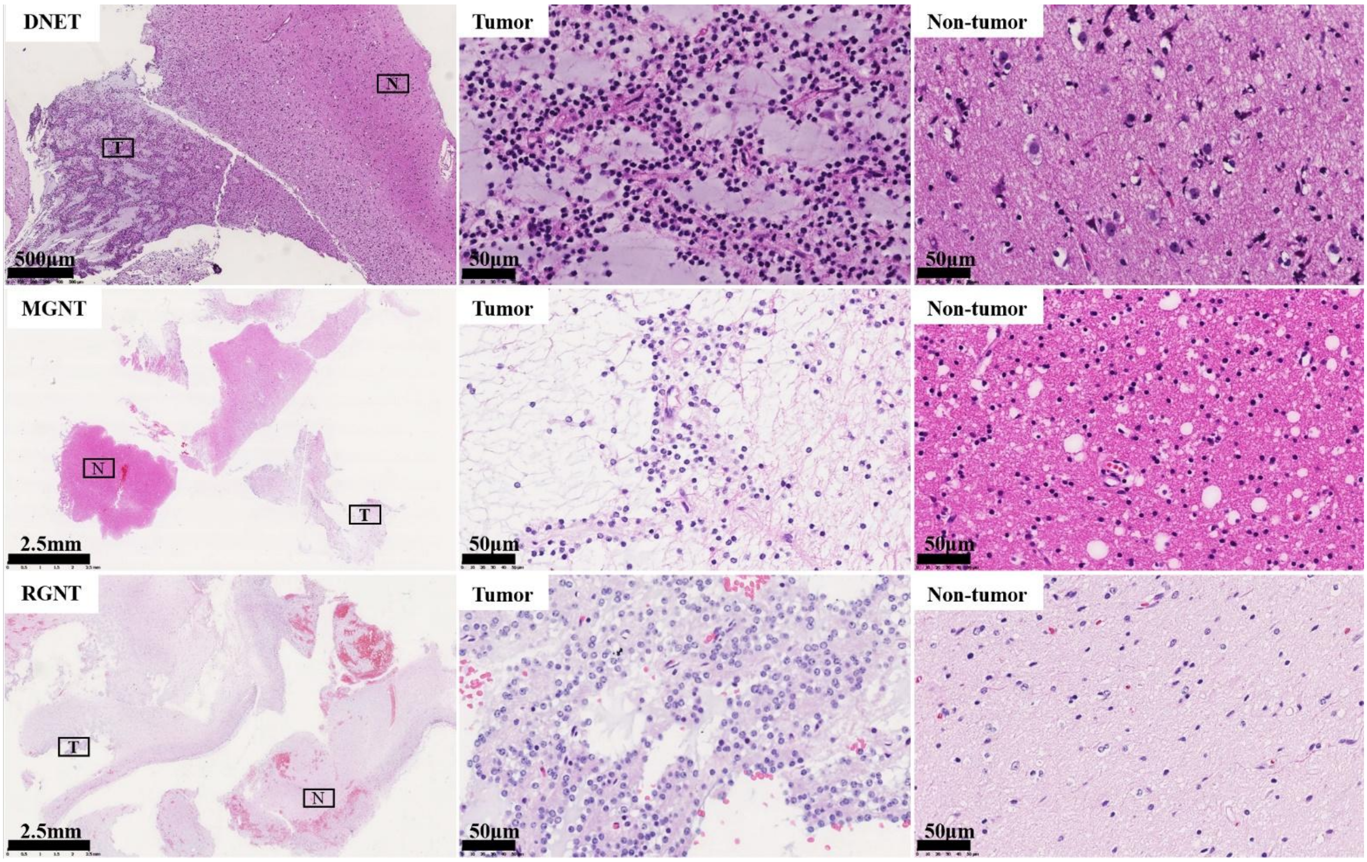

Supplementary Figure 1. Morphological features of the representative DNET, MGNT, and RGNT samples examined in this study.

In the tumor region (T), OLC images of DNET-6, MGNT-1, and the neurocytic component of RGNT-1 are shown. Non-tumor (N) regions are derived from the same section (x12.5, scale bar: 2.5 mm; x400, scale bar: 50 µm).

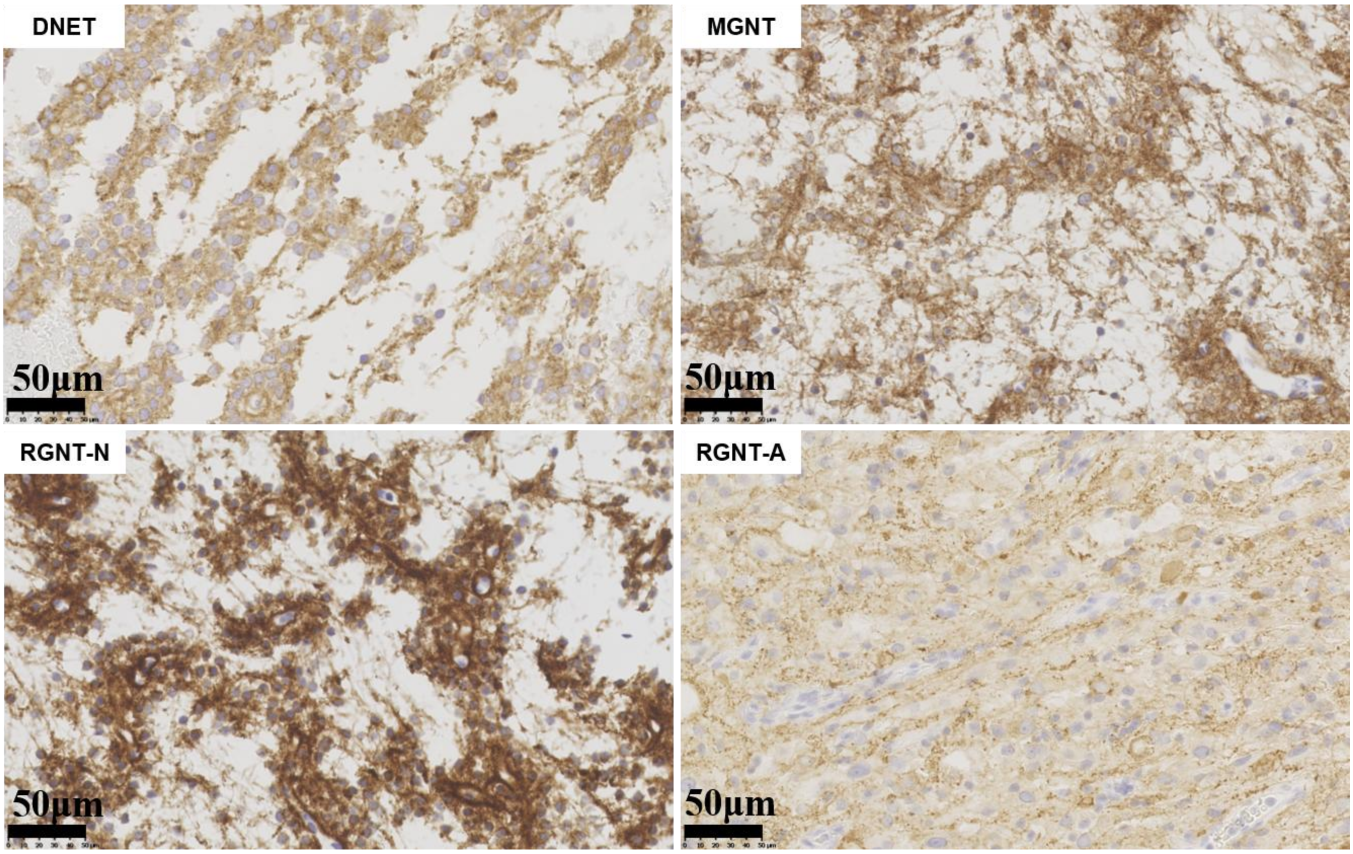

Supplementary Figure 2. SYN stainings in LGNTs unlikely indicate neuronal component. Representative SYN staining images in DNET (DNET-9), MGNT(MGNT-2), and the neurocytic (RGNT-N) and astrocytic (RGNT-A) regions of RGNT (RGNT-1) are shown. Granular SYN staining was observed throughout the background neuropil in all three types of LGNT, especially at the center of neurocytic rosettes and perivascular pseudorosettes in the RGNT samples (x400, scale bar: 50 μm).

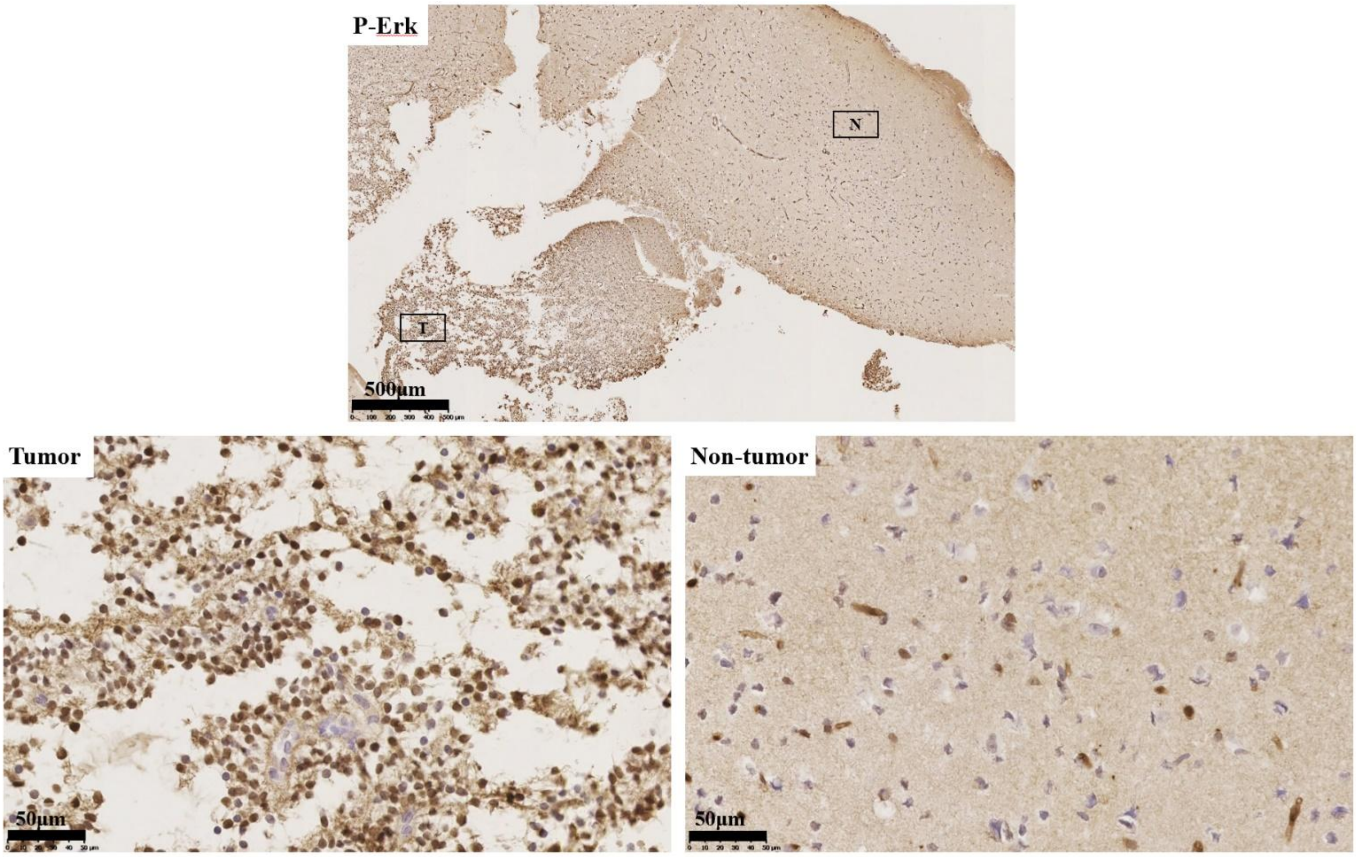

Supplementary Figure 3. P-Erk staining images in the tumor and non-tumor regions in a representative DNET sample.

In the tumor (T) region, OLCs were positively stained with P-Erk; while in non-tumor (N) region of the same section, some scattered endothelial cells were positively stained with P-Erk (x50, scale bar: 500 μm; x400, scale bar: 50 μm). Images are derived from DNET-6.

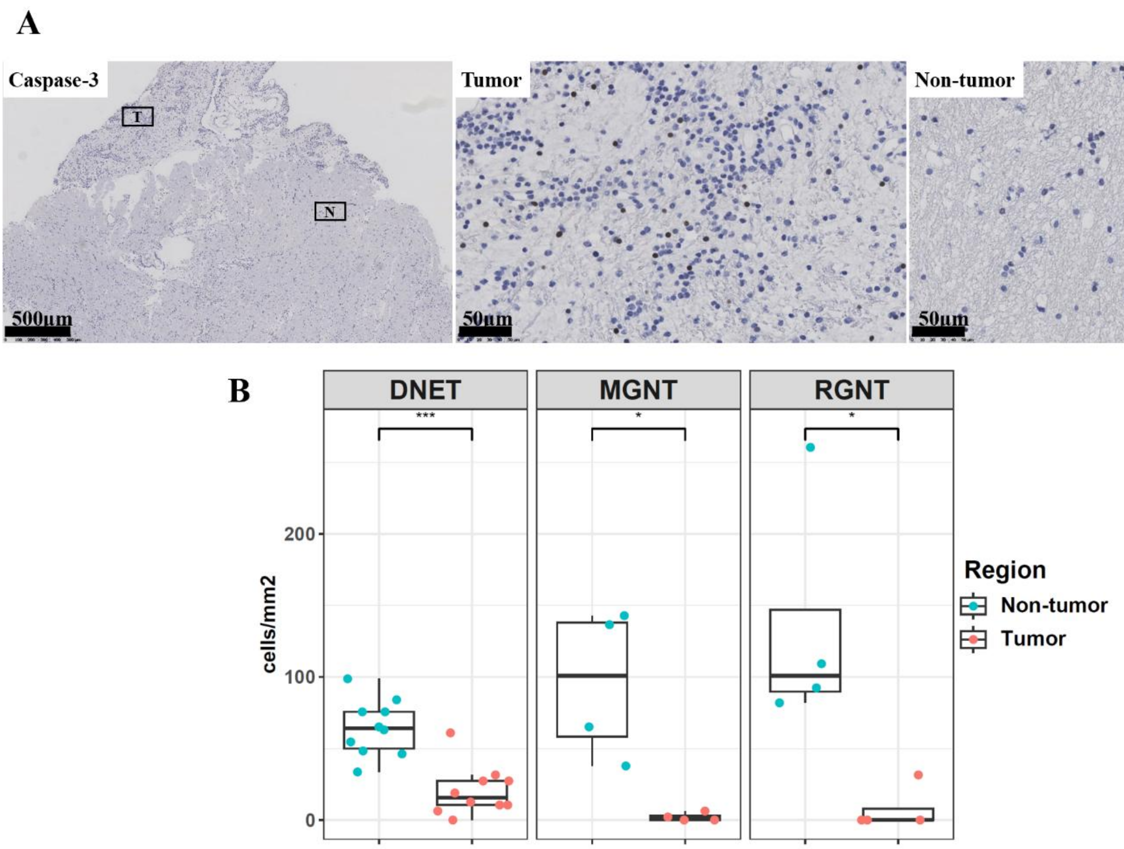

Supplementary Figure 4. Caspase-3 staining in the tumor and non-tumor regions in LGNTs.

(A) Images of caspase-3 staining in the tumor (T) region and non-tumor (N) region of a representative sample. Few scattered OLCs were positively stained for Caspase-3 in the tumor region, while negative staining was observed in the non-tumor region (x50, scale bar: 500 μm; x400, scale bar: 50 μm). Data are derived from MGNT-1.

(B) Quantification data of caspase-3 staining results in the three LGNT subtypes examined. DNET, N = 10, ***: paired t test, t = 5.1282, p = 0.0006211; MGNT, N = 4, *: paired t test, t = 3.5356, p = 0.03849; RGNT, N = 4, *: paired t test, t = 3.7602, p = 0.03288. Each data-point represents the average number of positive cells/mm^2 in an individual sample.

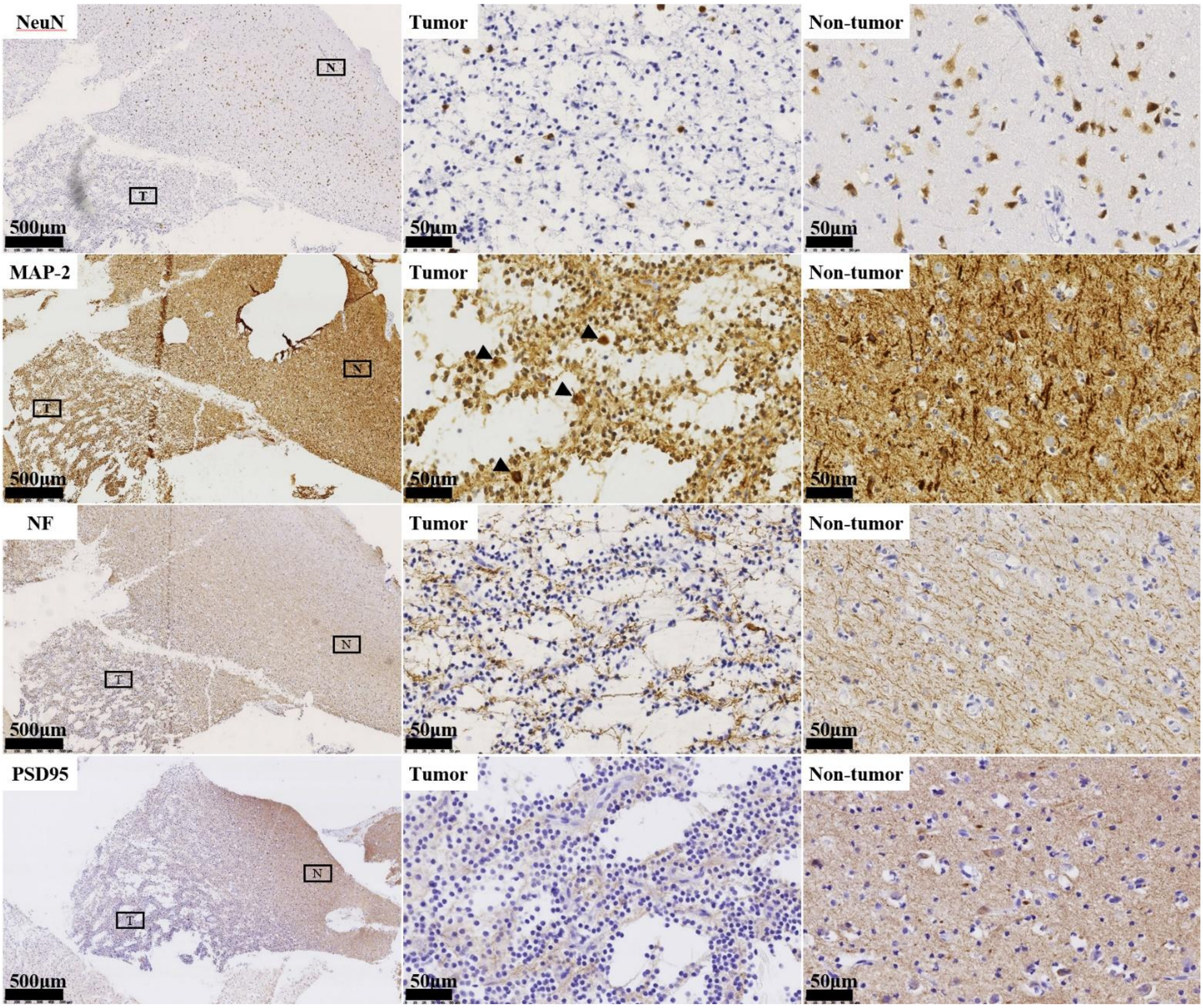

Supplementary Figure 5. Pervasive axon components in LGNTs.

Representative IHC images of NeuN, MAP-2, NF, and PSD95 stainings in the tumor (T) region and non-tumor (N) region of DNET-6 are shown. NeuN and MAP-2 stained neurons were scatted in the tumor region (arrows indicate the MAP-2 positively stained neurons), while NF and PSD95 stained axons were widely dispersed. There were more NeuN and MAP-2 stained neurons, and more NF and PSD95 stained axons in the non-tumor region (cortex) of the same section (x50, scale bar: 500 μm; x400, scale bar: 50 μm).

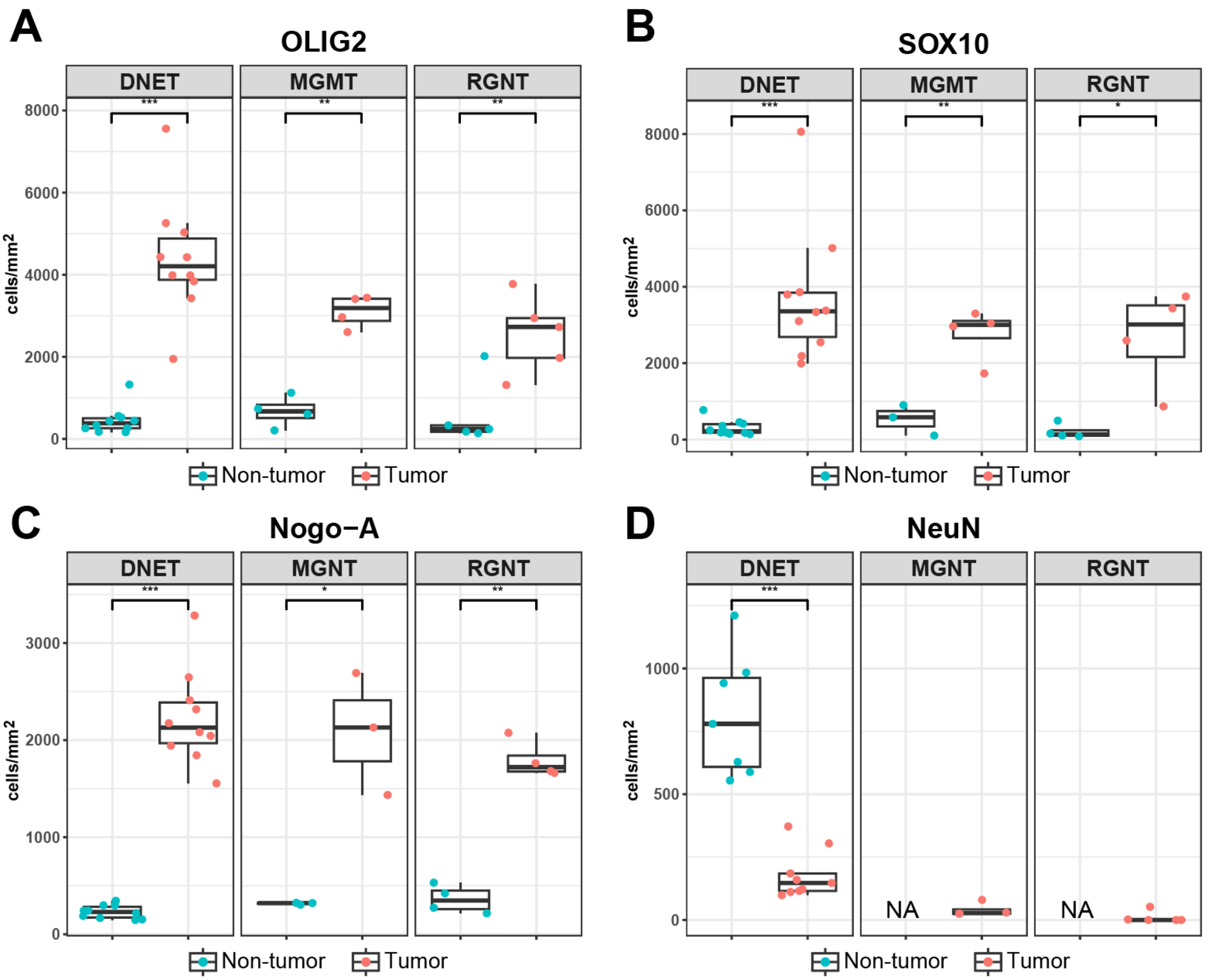

Supplementary Figure 6. Tumor cells expressed oligodendrocyte lineage markers but not neuronal marker.

The number of cells positively stained with pan-oligodendrocyte lineage markers OLIG2 (A) or SOX10 (B), or with developing oligodendrocyte marker Nogo-A (C), or with neuronal marker NeuN (D) in the tumor regions and non-tumor regions are shown. Because of the location of the onset of the disease away from the cortex, no areas containing normal mature neuronal cells were found on the slides of MGNT and RGNT. (A) OLIG2: DNET, N = 10, ***: paired t test, t = -8.5631, p = 1.28e-05; MGNT, N = 4, **: paired t test, t = -7.6939, p = 0.004563; RGNT, N = 5, **: paired t test, t = -6.0765, p = 0.003706. (B) SOX10: DNET, N = 10, ***: paired t test, t = -6.281, p = 0.000144; MGNT, N = 4 (the non-tumor region of MGNT_4 is unavailable), **: t test, t = -5.3131, p = 0.003519; RGNT, N = 4, *: paired t test, t = -4.1362, p = 0.02565. (C) Nogo-A: DNET, N = 10, ***: paired t test, t = -13.118, p = 3.594e-07; MGNT, N = 3, *: paired t test, t = -4.8708, p = 0.03966; RGNT, N = 4, **: paired t test, t = -9.0284, p = 0.002869. (D) NeuN: DNET, N = 10, ***: t test, t = 6.4982, p = 0.0002586; MGNT, N = 4; RGNT, N = 5. Each data-point represents the average number of positive cells/mm^2 in an individual sample. Detailed data was included in Supplementary Table 3.

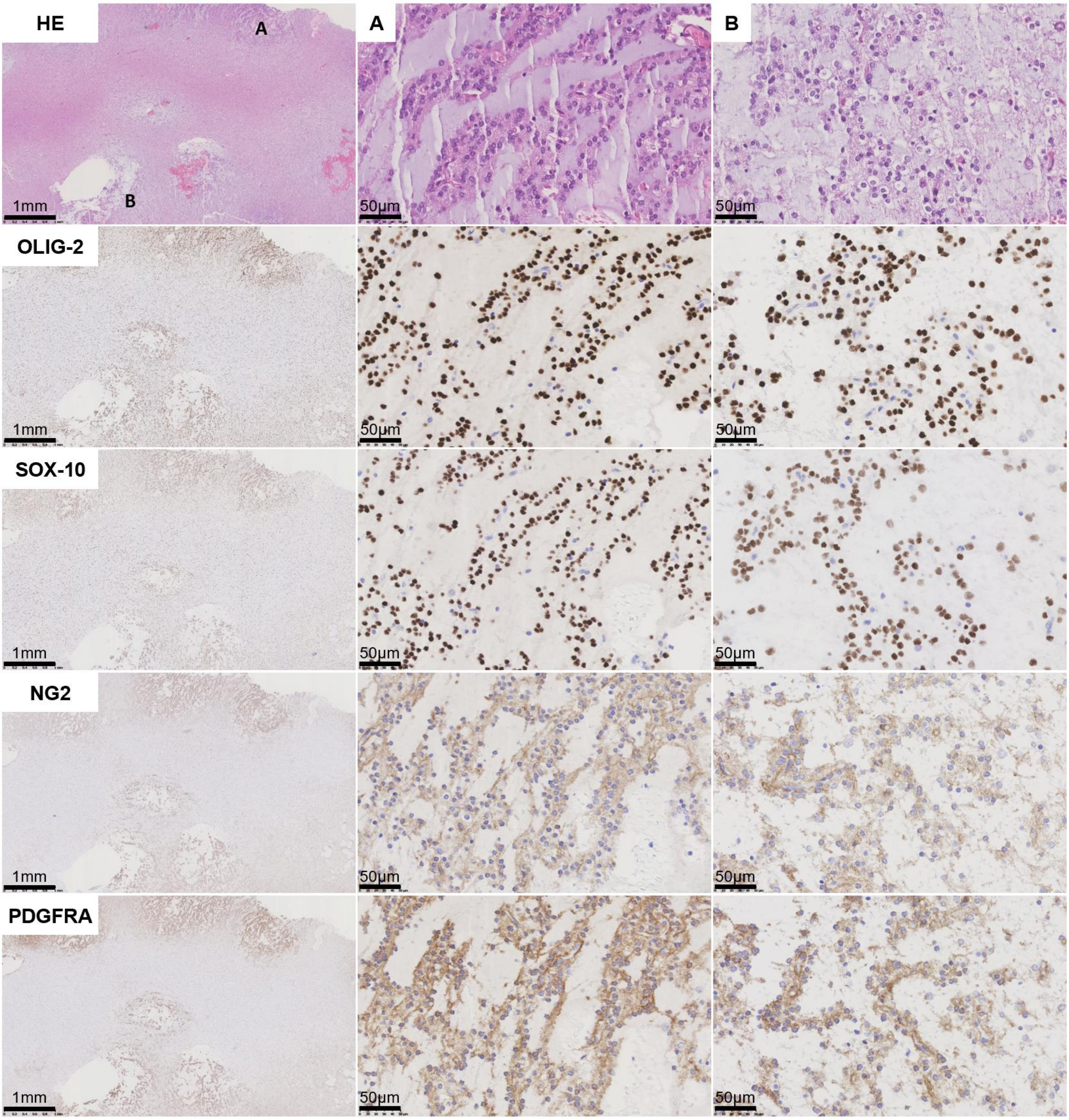

Supplementary Figure 7. Spatial maintenance of the expression profile of oligodendrocyte-lineage markers and OPC markers in different nodules within the same DNET sample.

HE and IHC images in different intracortical nodules of a representative DNET (DNET-10) are shown. Strong and consistent positive stainings of OLIG2, SOX10, NG2 and PDGFRA in both nodule A and B of this DNET were observed (x25, scale bar: 1 mm; x400, scale bar: 50 μm), Data shown are representative of nine samples examined.

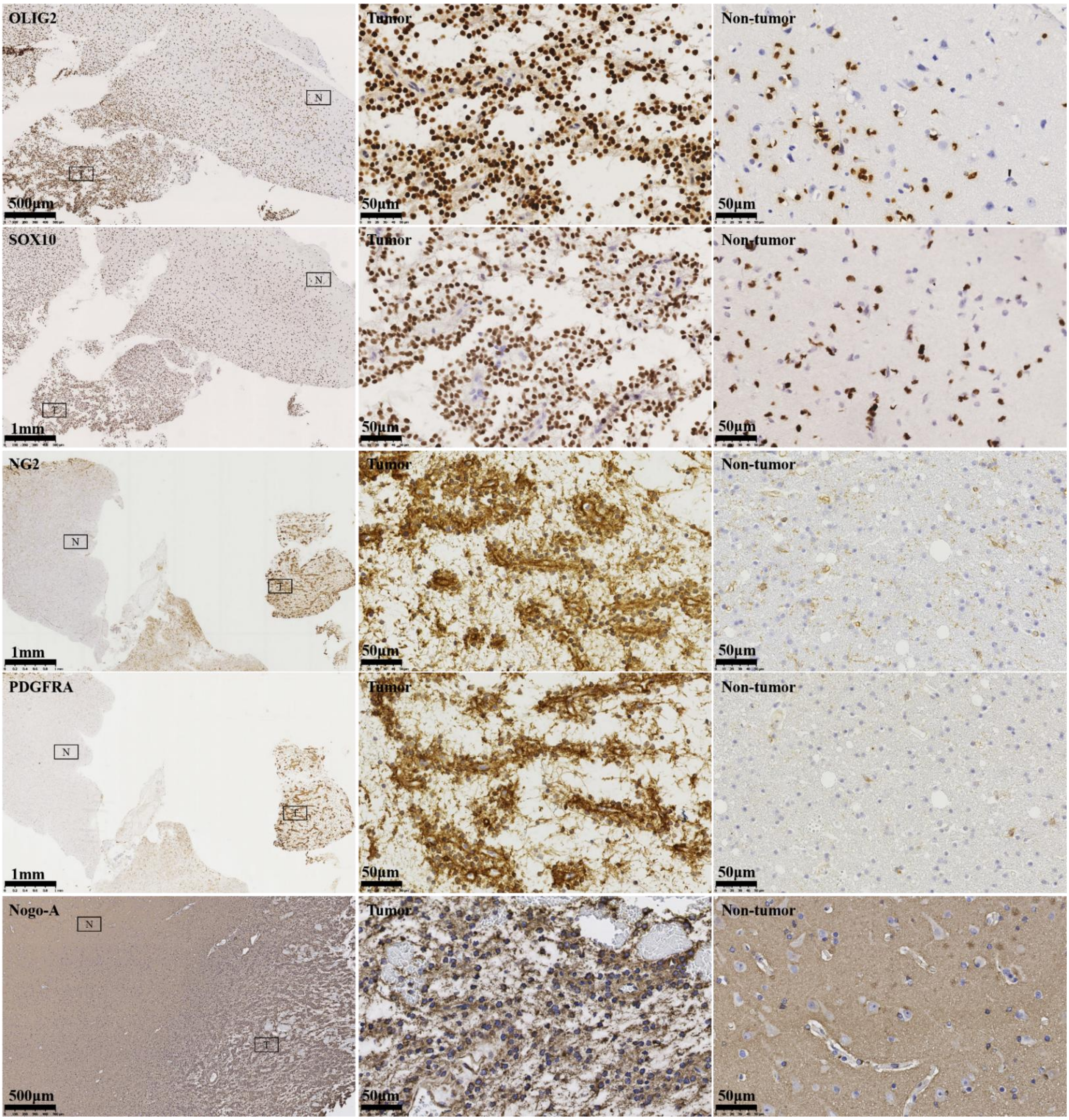

Supplementary Figure 8. Expression pattern of pan-oligodendrocyte lineage markers and OPC markers in LGNTs.

Representative IHC images in the tumor (T) and non-tumor (N) region of OLIG2 and SOX10 stainings in DNET-6, NG2 and PDGFRA stainings in MGNT-1, and Nogo-A staining of DNET-9 are shown. OLCs in tumor regions were strongly stained with OLIG2, SOX10, NG2, PDGFRA, and Nogo-A. Scatter or few OLIG2, SOX10, NG2, PDGFRA, and Nogo-A positive cells were found in non-tumor region (cortex or white matter) in the same section (x25, scale bar: 1 mm; x50, scale bar: 500 µm; x400, scale bar: 50 µm).

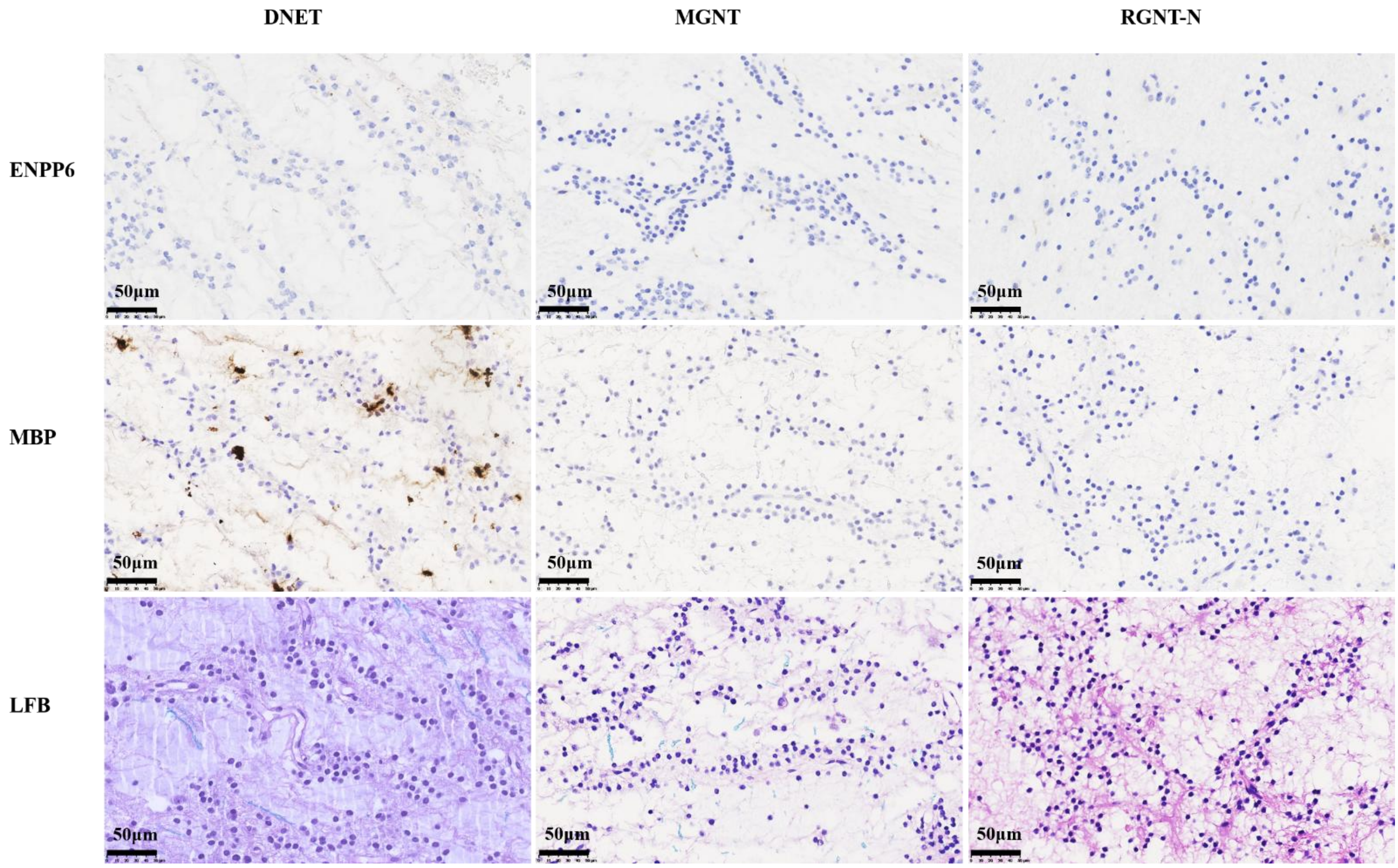

Supplementary Figure 9. Negative stainings of ENPP6, MBP, and LFB in LGNT samples.

Representative ENPP6, MBP, and LFB staining images in DNET (DNET-4), MGNT (MGNT-3), and the neurocytic (RGNT-N) region of RGNT (RGNT-1) are shown. Stainings for ENPP6, MBP, and LFB in tumor cells were negative in all cases examined. Few residual myelin was positively stained with MBP in DNET, and with LFB in DNET and MGNT (x400, scale bar: 50 μm).

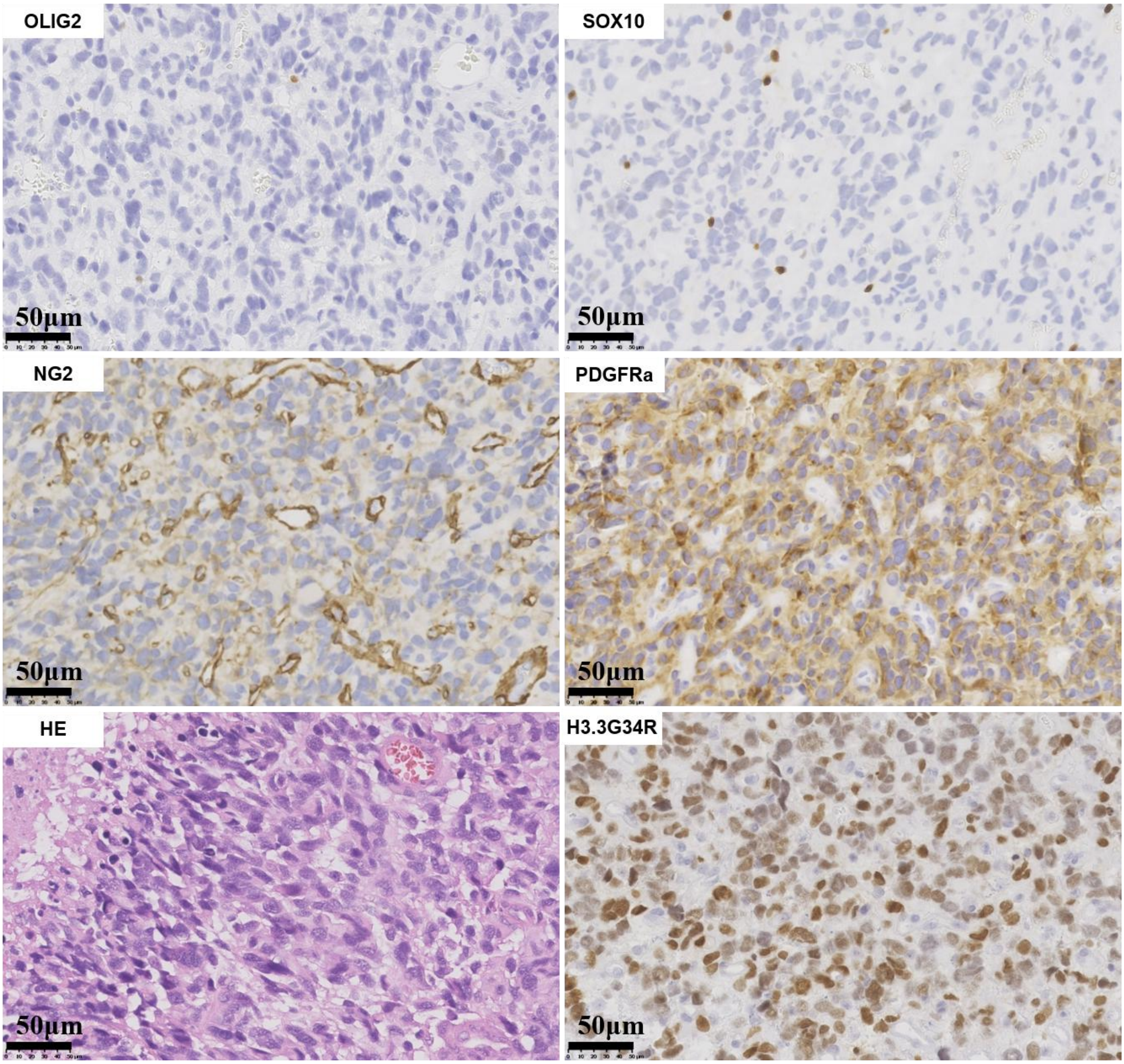

Supplementary Figure 10. Staining patterns of oligodendrocyte-lineage markers and OPC markers in the control DHG samples.

Representative images of stainings of oligodendrocyte-lineage markers (OLIG2 and SOX10) and OPC markers (NG2 and PDGFRA) in control DHG samples with their classical HE staining and mutation-specific antibody staining against H3.3 p.G35R (G34R)-mutant protein (H3.3G34R) are shown. OLIG2 and SOX10 staining were negative in tumor cells but were positive in few reactive glial cells. NG2 staining was strongly positive in vessel endothelial cells, which were scattered among tumor cells. PDGFRA staining was diffusely positive. HE staining showed characteristic morphological features of embryonic tumors in central nervous system. Diffusely positive nuclear staining of H3.3G34R mutant protein was observed in tumor cells (x 400, scale bar: 50 μm).

Supplementary Table 1. List of 425 cancer-related genes.

ABCB1(MDR1)	CDC73	ERCC2	IDH2	MTOR	PRKACA	SOX14
ABCB4	CDH1	ERCC3	IFNG	MUTYH	PRKACG	SOX2
ABCC2(MRP2)	CDK10	ERCC4	IFNGR1	MYC	PRKAR1A	SOX21
ADH1A	CDK12	ERCC5	IGF1R	MYCL	PRKCI	SPOP
ADH1B	CDK4	ESR1	IGF2	MYCN	PRKDC	SPRY4
ADH1C	CDK6	ETV1	IKBKE	MYD88	PRSS1	SRC
AIP	CDK8	ETV4	IKZF1	MYH9	PRSS3	SRY
AKT1	CDKN1A	ETV6	IL7R	NAT1	PTCH1	STAG2
AKT2	CDKN1B	EWSR1	INPP4B	NBN	PTEN	STAT3
AKT3	CDKN1C	EXT1	IRF2	NCOR1	PTK2	STK11
ALDH2	CDKN2A	EXT2	JAK1	NF1	PTPN11	STMN1
ALK	CDKN2B	EZH2	JAK2	NF2	PTPN13	STT3A
AMER1	CDKN2C	FANCA	JAK3	NFE2L2	PTPRD	SUFU
APC	CEBPA	FANCC	JARID2	NFKBIA	QKI	TAP1
AR	CEP57	FANCD2	JUN	NKX2-1	RAC1	TAP2
ARAF	CHD4	FANCE	KDM5A	NKX2-4	RAC3	TEK
ARID1A	CHEK1	FANCF	KDM6A	NOTCH1	RAD50	TEKT4
ARID1B	CHEK2	FANCG	KDR(VEGFR2)	NOTCH2	RAD51	TERC
ARID2	CREBBP	FANCI	KEAP1	NOTCH3	RAD51B	TERT
ARID5B	CRKL	FANCL	KIF1B	NPM1	RAD51C	TET2
ASCL4	CSF1R	FANCM	KIF5B	NQO1	RAD51D	TGFBR2
ASXL1	CTCF	FAT1	KIT	NRAS	RAD54L	THADA
ATF1	CTLA4	FBXW7	KITLG	NRG1	RAF1	TMEM127
ATIC	CTNNB1	FGF19	KLLN	NSD1	RARA	TMPRSS2
ATM	CUL3	FGFR1	KMT2A(MLL)	NTRK1	RARG	TNFAIP3
ATR	CUX1	FGFR2	KMT2B	NTRK2	RASGEF1A	TNFRSF11A
ATRX	CXCR4	FGFR3	KMT2C	NTRK3	RB1	TNFRSF14
AURKA	CYLD	FGFR4	KMT2D(MLL2)	PAK3	RECQL4	TNFRSF19
AURKB	CYP19A1	FH	KRAS	PALB2	RELN	TNFSF11
AXIN2	CYP2A13	FLCN	LHCGR	PALLD	RET	TOP1
AXL	CYP2A6	FLT1(VEGFR1)	LMO1	PARK2	RHOA	TOP2A
B2M	CYP2A7	FLT3	LRP1B	PARP1	RICTOR	TP53
BAD	CYP2B6*6	FLT4	LYN	PARP2	RNF43	TP63
BAI3	CYP2C19*2	FOXA1	LZTR1	PAX5	ROS1	TPMT
BAK1	CYP2C9*3	FOXP1	MAP2K1(MEK1)	PBRM1	RPTOR	TSC1
BAP1	CYP2D6	FRG1	MAP2K2(MEK2)	PDCD1(PD1)	RRM1	TSC2
BARD1	CYP3A4*4	GATA1	MAP2K4	PDCD1LG2(PD-L2)	RUNX1	TSHR
BAX	CYP3A5	GATA2	MAP3K1	PDE11A	RUNX1T1	TTF1

BCL2	DAXX	GATA3	MAP3K4	PDGFRA	SBDS	TUBB3
BCL2L11(BIM)	DDR2	GATA4	MAP4K3	PDGFRB	SDC4	TUBB4A
BCR	DENND1A	GATA6	MAX	PDK1	SDHA	TUBB4B
BIRC3	DHFR	GNA11	MCL1	PGR	SDHB	TUBB6
BLM	DICER1	GNAQ	MDM2	PHOX2B	SDHC	TYMS
BMPR1A	DLL3	GNAS	MDM4	PIK3C3	SDHD	U2AF1
BRAF	DNMT3A	GRIN2A	MECOM	PIK3CA	9-Sep	UGT1A1
BRCA1	DPYD	GRM3	MED12	PIK3R1	SETBP1	VAMP2
BRCA2	DUSP2	GRM8	MEF2B	PIK3R2	SETD2	VEGFA
BRD4	EGFR	GSTM1	MEN1	PKHD1	SF3B1	VHL
BRIP1	EML4	GSTM4	MET	PLAG1	SGK1	WAS
BTG2	EP300	GSTM5	MGMT	PLK1	SLC34A2	WISP3
BTK	EPAS1	GSTP1	MITF	PMS1	SLC3A2	WRN
BUB1B	EPCAM	GSTT1	MLH1	PMS2	SLC7A8	WT1
c11orf30	EPHA2	HDAC2	MLH3	POLD1	SMAD2	XPA
CASP8	EPHA3	HDAC9	MLLT1	POLD3	SMAD3	XPC
CBL	EPHA5	HGF	MLLT3	POLE	SMAD4	XRCC1
CBLB	EPHB2	HLA-A	MLLT4	POLH	SMAD7	YAP1
CCND1	ERBB2(HER2)	HNF1A	MPL	POT1	SMARCA4	ZNF2
CCNE1	ERBB2IP	HNF1B	MRE11A	PPARD	SMARCB1	ZNF217
CD274(PD-L1)	ERBB3	HRAS	MSH2	PPP2R1A	SMO	ZNF703
CD74	ERBB4	HSD3B1	MSH6	PRDM1	SOS1	CDA
ERCC1	IDH1	MTHFR	PRF1	SOX1		

Supplementary Table 2. Histological and immunohistochemical features of DNET, MGNT, and RGNT samples examined.

Case ID	GFAP	OLIG2	S-100	MAP-2	NeuN	NF	SYN	ATRX	TP53	CD34	IDH1 (R132H)	H3K27M	BRAF V600E	Ki-67
**DNET-1**	0	4+	4+	4+	2+	0	background	no loss expression	0	0	0	0	0	1%
**DNET-2**	0	4+	4+	4+	2+	0	background	no loss expression	0	0	0	0	0	1%
**DNET-3**	0	4+	4+	4+	2+	0	background	no loss expression	0	0	0	0	0	3-4%
**DNET-4**	1+	4+	4+	4+	2+	0	background	no loss expression	0	0	0	0	0	1-2%
**DNET-5**	2+	4+	4+	4+	2+	0	background	no loss expression	0	1+	0	0	0	1-2%
**DNET-6**	0	4+	4+	4+	2+	0	background	no loss expression	0	0	0	0	0	1-3%
**DNET-7**	1+	4+	4+	4+	2+	0	background	no loss expression	0	0	0	0	0	2-3%
**DNET-8**	0	4+	4+	4+	2+	0	background	no loss expression	0	1+	0	0	0	1-2%
**DNET-9**	0	4+	4+	4+	2+	0	background	no loss expression	0	0	0	0	0	3%
**DNET-10**	0	4+	4+	4+	2+	0	background	no loss expression	0	0	0	0	0	1-3%

**MGNT-1**	0	4+	4+	4+	1+	0	background	no loss expression	0	0	0	0	0	2%
**MGNT-2**	0	4+	4+	4+	1+	0	background	no loss expression	0	0	0	0	0	3%
**MGNT-3**	0	4+	4+	4+	1+	0	background	no loss expression	0	0	0	0	0	3%
**MGNT-4**	0	4+	4+	4+	1+	0	background	no loss expression	0	0	0	0	0	2%
**RGNT-1**	2+(AC); 0 (NC)*	0 (AC); 4+(NC)	4+	4+	1+	0	background	no loss expression	0	0	0	0	0	2%
**RGNT-2**	2+(AC); 0 (NC)	0 (AC); 4+(NC)	4+	4+	0	0	background	no loss expression	0	0	0	0	0	2%
**RGNT-3**	3+(AC); 0 (NC)	0 (AC); 4+(NC)	4+	4+	0	0	background	no loss expression	0	0	0	0	0	2%
**RGNT-4**	2+(AC); 0 (NC)	0 (AC); 4+(NC)	4+	4+	0	0	background	no loss expression	0	0	0	0	0	2%
**RGNT-5**	2+(AC); 0 (NC)	0 (AC); 4+(NC)	4+	4+	0	0	background	no loss expression	0	3+	0	0	0	4%

The frequency of immunopositive tumor cells was semi-quantitatively assessed as described in the Materials and Methods.
*AC, astrocytic component; NC, neurocytic component.

Supplementary Table 3. Densities of cells positively stained with the oligodendrocyte lineage markers or neuronal marker in individual samples.

Case ID	Region	PDGFRA (cell/mm2)	NG2 (cell/mm2)	SOX10 (cell/mm2)	OLIG2 (cell/mm2)	Nogo-A (cell/mm2)	NeuN (cell/mm2)
DNET_1	Tumor	1371.8 ±91.4	1386.6 ±136.9	1991.6 ±98.9	3426.5 ±508	1943.3 ±132.7	371.8 ±104.9
	Non-tumor	39.9 ±3.6	111.3 ±31.7	184.9 ±10.3	523.1 ±40.9	296.2 ±69.9	1210.1 ±181.3
DNET_2	Tumor	1445.4 ±168.6	1508.4 ±200.1	2187 ±177.7	1947.5 ±159.9	1844.5 ±74.8	304.6 ±76.6
	Non-tumor	71.4 ±15.1	48.3 ±9.2	187 ±21.7	163.9 ±22.6	243.7 ±78.2	588.2 ±41.6
DNET_3	Tumor	1857.1 ±384	2161.8 ±189.7	3859.2 ±169.3	4426.5 ±429.9	2081.9 ±392.2	184.9 ±35.7
	Non-tumor	273.1 ±56.2	86.1 ±40.5	453.8 ±73.5	445.4 ±151.6	344.5 ±60.3	NA
DNET_4	Tumor	2081.9 ±134.8	1619.7 ±216.7	2548.3 ±684	4430.7 ±431.3	2647.1 ±762.2	115.5 ±29.3
	Non-tumor	44.1 ±7	71.4 ±12.6	153.4 ±38.7	327.7 ±36.1	153.4 ±20.9	983.2 ±140.9
DNET_5	Tumor	1863.4 ±164.4	2369.7 ±245.2	5014.7 ±666	5256.3 ±167.8	2411.8 ±330.6	111.3 ±58.9
	Non-tumor	157.6 ±52.9	NA	142.9 ±29.7	260.5 ±46.4	189.1 ±19.3	554.6 ±60.9
DNET_6	Tumor	2670.2 ±477.2	2672.3 ±714.4	8058.8 ±2745.7	7556.7 ±888.4	3283.6 ±823.8	159.7 ±39.9
	Non-tumor	264.7 ±25.6	117.6 ±75.9	416 ±43.5	434.9 ±82	298.3 ±54.9	NA
DNET_7	Tumor	1586.1 ±117	1516.8 ±288.5	3798.3 ±273.9	5031.5 ±640.8	2317.2 ±334.6	147.1 ±44.7
	Non-tumor	52.5 ±15	58.8 ±24.5	357.1 ±47	558.8 ±74.8	168.1 ±79.3	941.2 ±56.4
DNET_8	Tumor	1590.3 ±171.8	1535.7 ±211.7	3338.2 ±146.7	3983.2 ±205.8	1554.6 ±238.4	121.8 ±27.6
	Non-tumor	NA	84 ±26.6	178.6 ±83.7	172.3 ±30.6	216.4 ±33.8	628.2 ±57.1
DNET_9	Tumor	1880.3 ±244.1	1298.3 ±138.8	3100.8 ±160.4	3840.3 ±423.9	2044.1 ±298.4	98.7 ±16.1
	Non-tumor	105 ±32.3	176.5 ±27.2	243.7 ±42.8	262.6 ±58.3	147.1 ±45.4	779.4 ±66.8

**DNET_10**	**Tumor**	1573.5 ±352.3	1344.5 ±180.5	3378.2 ±460.3	3985.3 ±232.4	2174.4 ±276.1	NA
	**Non-tumor**	92.4 ±13.3	16.8 ±13.3	773.1 ±126.2	1325.6 ±151.8	243.7 ±44.9	NA
**MGNT_1**	**Tumor**	1224.8 ±220	1552.5 ±385.3	2964.3 ±269.8	2966.4 ±576.1	1434.9 ±171.9	29.4 ±4.2
	**Non-tumor**	134.5 ±25.9	79.8 ±9.4	903.4 ±93.3	1123.9 ±168.6	321.4 ±43	NA
**MGNT_2**	**Tumor**	1890.8 ±251.7	1531.5 ±185.9	3298.3 ±278.4	3437 ±785	2691.2 ±876	79.8 ±45.1
	**Non-tumor**	81.9 ±40.5	113.4 ±59.6	584 ±337.1	737.4 ±256.3	323.5 ±47.7	NA
**MGNT_3**	**Tumor**	1632.4 ±294.3	1464.3 ±251.8	3044.1 ±433.5	3409.7 ±870.9	2130.3 ±545.6	25.2 ±11.9
	**Non-tumor**	37.8 ±12.6	58.8 ±26.6	105 ±27.6	208 ±81.1	304.6 ±36.3	NA
**MGNT_4**	**Tumor**	NA	NA	1733.2 ±330.7	2602.9 ±1100.7	NA	25.2 ±8.4
	**Non-tumor**	NA	NA	NA	607.1 ±241.8	NA	NA
**RGNT_1**	**Tumor**	1567.2 ±221.9	1264.7 ±306.5	2592.4 ±259.7	2726.9 ±681.8	1661.8 ±48.4	52.5 ±16.1
	**Non-tumor**	21 ±9.4	42 ±39	159.7 ±144.5	140.8 ±32.2	422.3 ±59.5	NA
**RGNT_2**	**Tumor**	1575.6 ±481.7	1321.4 ±200	3434.9 ±583.2	2943.3 ±806.7	2075.6 ±119.4	0
	**Non-tumor**	6.3 ±3.6	94.5 ±16.1	107.1 ±76.9	176.5 ±77.2	216.4 ±38.7	NA
**RGNT_3**	**Tumor**	1184.9 ±120.5	1090.3 ±122.8	3745.8 ±741.4	3773.1 ±713.3	1680.7 ±386	0
	**Non-tumor**	18.9 ±7	132.4 ±58.6	495.8 ±112	2018.9 ±159.9	531.5 ±94.4	NA
RGNT_4	**Tumor**	NA	NA	NA	1315.1 ±538.5	NA	2.1 ±3.6
	**Non-tumor**	NA	NA	NA	331.9 ±30.6	NA	NA
RGNT_5	**Tumor**	785.7 ±147.3	1130.3 ±127.5	867.6 ±244.1	1974.8 ±222.6	1762.6 ±86.4	0
	**Non-tumor**	2.1 ±3.6	33.6 ±10.3	88.2 ±56.2	241.6 ±22.5	273.1 ±38.3	NA

The data shown are the average number of cells under 4 microscopic fields in each sample, presented in average ±standard deviation format.

Supplementary Table 4. The gene lists used in GSEA.

NSC	OPC	NFO	MO	Astro
EFEMP1	PDGFRA	GP1BB	GJB1	HGF
ITGA6	CSPG4	FYN	NDRG1	AQP4
F2RL1	CD9	NFASC	ASPA	ITIH3
DPYSL3	CNP	PIK3R3	EFHD1	BMPR1B
AQP4	GPR17	BMP4	ITGB4	ITGA7
EPHX2	PTPRZ1	MCL1	MBP	GRM3
ID4	HES1	CYFIP2	MAL	SLC14A1
ANGPT1	ID2	SLC12A2	MOG	PHKG1
AK1	MYT1	ITPR2	SLCO3A1	CBS
BBOX1	NKX2-2	CHN2	APOD	ALDH1L1
DLEC1	OLIG2	PPP2R3A	GSN	CTH
DNAH9	LNX1	GLRB	TBC1D9B	FMO1
COL21A1	MMP15	SEMA5A	NOL3	SLC6A11
RARRES2	KCNK1	FAM3C	CENPB	FGFR3
DNALI1	CHRNA4	NKX2-2	RFTN1	SLC4A4
ECM2	PID1	OLIG2	CRYAB	EGFR
ANXA1	UGDH	SOX3	KIF5A	KCNN3
CD24	RLBP1	MYCL1	TRAK2	PTX3
CETN2	SDC3	CD9	POU3F1	TNC
RNASE4	SOX9	CNP	NFE2L3	SOX9
CD99	RTN1	MAG	CD9	ABCD2
ITGAV	FGF12	MAL	GALC	FZD10
PLTP	FABP7	PLP1	CNP	LRIG1
	ISG15	SMARCA4	MAG	MLC1
	TRO	MYT1	MOBP	GLI1
		ZNF24	PLP1	GLI2
		ZNF536	SMARCA4	GLI3
			MYT1	SOX9
			C11orf9	PAX6
			OLIG2	
			ZNF24	
			ZNF536	
			MBP	
			MAL	
			MOG	

The genes listed above were the intersection between the origin signature genes and the genes actually detected in GSE60898.
